# Supplementary figures and images for: TRIM25 and ZAP target the Ebola virus ribonucleoprotein complex to mediate interferon-induced restriction
Source: PLoS Pathog. 2022 May 9;18(5):e1010530. doi: 10.1371/journal.ppat.1010530 (PMC9119685; doi:10.1371/journal.ppat.1010530)

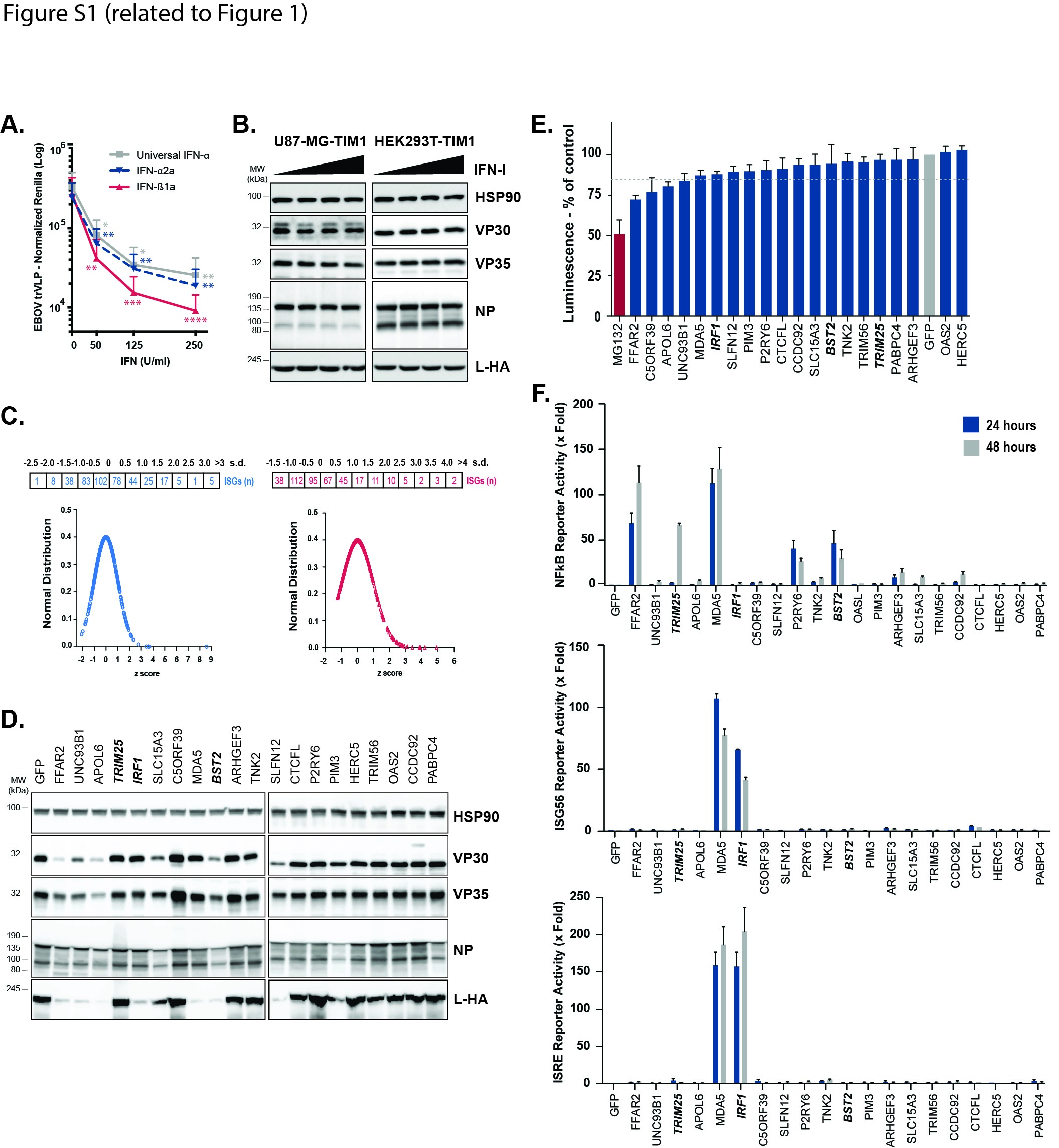

Supplement: S1 Fig — EBOV trVLP assay and characterization of top candidate inhibitory ISGs. (A) U87-MG-TIM1 cells were transfected with plasmids expressing EBOV’s RNP components and pre-treated with increasing amounts of Universal type-I IFN-α, IFN-α2a or IFN-ß1a, 16–24 hours prior to EBOV trVLP infection. Reporter activities were measured 24 hours later. (B) U87-MG-TIM1 and HEK293T-TIM1 cells were transfected with plasmids expressing EBOV NP, VP35, VP30 and a HA-tagged version of L-polymerase, before being treated with increasing amounts of IFN-I prior to EBOV trVLP infection as in Fig 1B. Cells were lysed 24 hours post-transfection and lysates analysed by western blot for the expression of the EBOV RNP proteins and HSP90. (C) ISG Screen Validation. Normal distribution of EBOV trVLP Rluc values in the presence of individually over-expressed ISGs on p1 (left panel, blue dots) and p2 (right panel, red dots) target cells, normalized to Fluc levels on the same well. The number (n) of ISGs within standard deviation (s.d. or z score) ranges is shown in the boxes. (D) HEK293T-TIM1 cells were transfected with plasmids expressing the EBOV RNP proteins together with selected ISGs. Cells lysates were analysed 48 hours later by western blot as in (B). (E) HEK293T-TIM1 cells were transfected with individual ISGs and EBOV RNP-expressing plasmids as in (D), and tested for cellular viability 48 hours later using Cell-Titer Glo luminescence-based assay (Promega). As positive control for toxicity (red bar), cells were treated for 48h with a concentration of MG132 (25μM) sufficient to reduce the ATP levels in the supernatant by 50 percent. Values are represented as percentage of luminescence obtained in control wells transfected with GFP (grey bar), and toxicity cut-off represented as a dashed line. (F) Fold activation of Firefly luciferase NF-kB, ISG56/IFIT1 or ISRE reporters (top, middle or bottom panels, respectively) in HEK293T cells transiently transfected with selected individual ISGs compared to [file ppat.1010530.s001.tif]

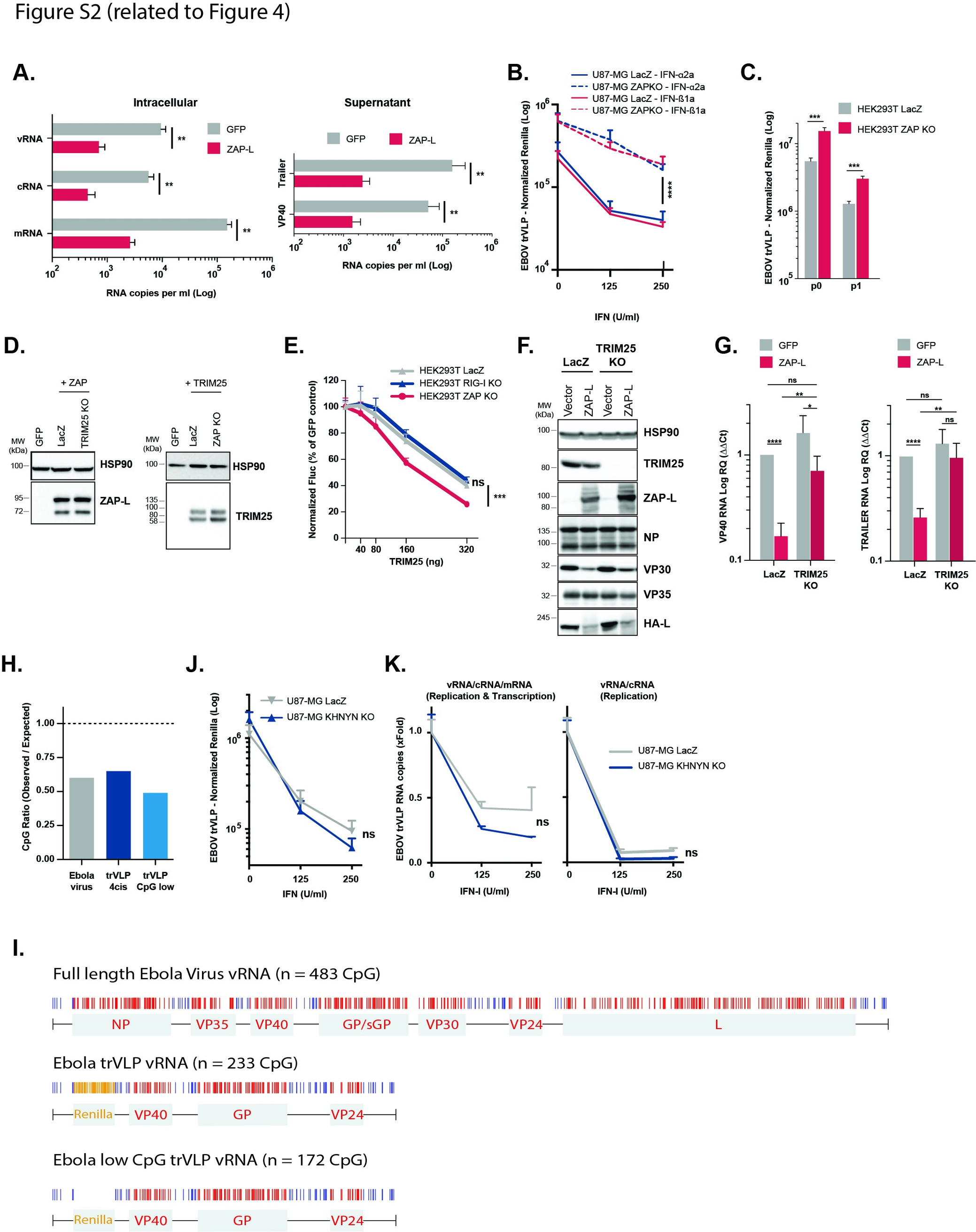

Supplement: S2 Fig — Overexpression of ZAP-L impacts on EBOV trVLP viral RNA levels. (A) Quantification of viral RNA transcripts present intracellularly (left panel) and in supernatants (right panel) of HEK293T-TIM1 cells transfected as in Fig 4A, and infected with a fixed amount of EBOV trVLPs. Strand-specific reverse transcription primers were used on total RNA extracted from cells to generate cDNAs for minigenomic RNA (vRNA), complementary RNA (cRNA), and mRNA, followed by RT-qPCR analysis. Random hexamer primers were used to generate cDNAs from total RNA extracted from supernatants, and qPCR analysis performed using primers targeting the 5’ trailer region of the 4cis minigenome or VP40 RNA. (B) EBOV trVLP reporter activities on U87-MG LacZ CRISPR-TIM1 (solid lines) and U87-MG ZAP CRISPR KO-TIM1 (dashed lines) target cells transfected with EBOV RNP proteins and pre-treated with increasing amounts of IFN-α2a (blue) or IFN-ß1b (red) prior to infection. (C) HEK293T LacZ CRISPR (grey) and HEK293T ZAP CRISPR KO (red) were used as producer cells (p0) for EBOV trVLPs, and reporter activities measured 48 hours post-transfection. Supernatants from p0 were used to infect HEK293T-TIM1 target cells (p1), and reporter activities determined 24 hours later. (D) Proteins levels of HSP90, ZAP and TRIM25 in lysates from Fig 4E were analysed by western blot on p1 cells harvest at time of infection. (E) Influenza A minigenome assay was performed in HEK293T LacZ CRISPR (grey), HEK293T ZAP CRISPR KO (red) and HEK293T RIG-I CRISPR KO (blue) cells transfected with Influenza polymerase components (NP, PB1, PB2 and PA) and increasing amounts of TRIM25. Normalized Fluc values are presented as percentage relative to a GFP control in each cell line. (F) HEK293T LacZ CRISPR, and HEK293T TRIM25 CRISPR KO cells were transfected with plasmids expressing the EBOV NP, VP35, VP30 proteins and a HA-tagged L Polymerase together with either pcDNA4 or ZAP-L. Cells lysates were analysed 48 hours later by western blot for th [file ppat.1010530.s002.tif]

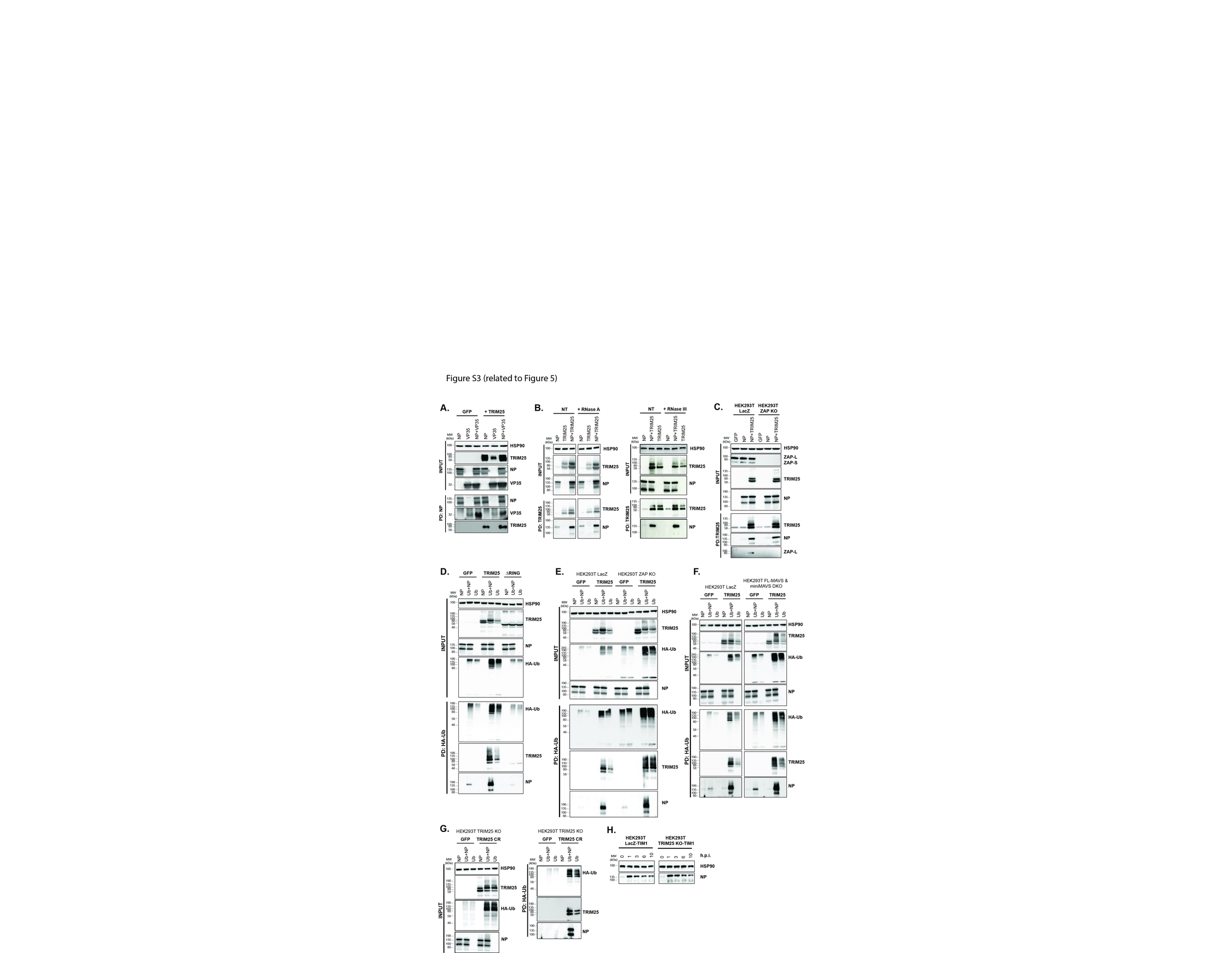

Supplement: S3 Fig — TRIM25-mediated ubiquitination of EBOV NP is independent of ZAP and MAVS. (A) Lysates of HEK293T-TIM1 cells transfected either with GFP or TRIM25, in combination with EBOV NP and/or EBOV VP35 were immunoprecipitated with an anti-NP antibody. Cellular lysates and pull-downs were analysed by western blot for HSP90, TRIM25, EBOV NP and VP35. (B) Lysates of HEK293T-TIM1 cells transfected either with EBOV NP and/or TRIM25 were left untreated (NT), or treated with RNase A (left panel) or RNAse III (right panel) prior to immunoprecipitation with an anti-TRIM25 antibody. Cellular lysates and pull-downs were analysed by western blot for HSP90, TRIM25 and EBOV NP. (C) Lysates of HEK293T LacZ CRISPR and HEK293T ZAP CRISPR KO cells transfected either with GFP or TRIM25 and/or EBOV NP were immunoprecipitated with anti-TRIM25 antibody. Cellular lysates and pull-down samples were analysed by western blot for HSP90, TRIM25, EBOV NP and ZAP. (D) HEK293T-TIM1 cells were transfected either with GFP, TRIM25 wild-type or TRIM25 ΔRING mutant, in combination with EBOV NP and/or a plasmid expressing a HA-tagged Ubiquitin (HA-Ub). Lysates from these cells were immunoprecipitated 48 hours later with an anti-HA antibody. Cellular lysates and pull-down samples were analysed by western blot for HSP90, TRIM25, NP and HA (ubiquitin). (E) HEK293T LacZ CRISPR and HEK293T ZAP CRISPR KO cells were transfected either with GFP or TRIM25, in combination with EBOV NP and/or HA-Ub. Lysates from these cells were immunoprecipitated 48 hours later with an anti-HA antibody. Cellular lysates and pull-down samples were analysed by western blot as in (D). (F) HEK293T LacZ control and HEK293T FL-MAVS/miniMAVS CRISPR DKO cells were transfected either with GFP or TRIM25 in combination with EBOV NP and/or HA-Ub. Lysates were immunoprecipitated with anti-HA antibody and analysed by western blotting as in (D). (G) HEK293T TRIM25 CRISPR KO cells were transfected either with GFP or TRIM25 CR in combination with EBOV NP [file ppat.1010530.s003.tif]

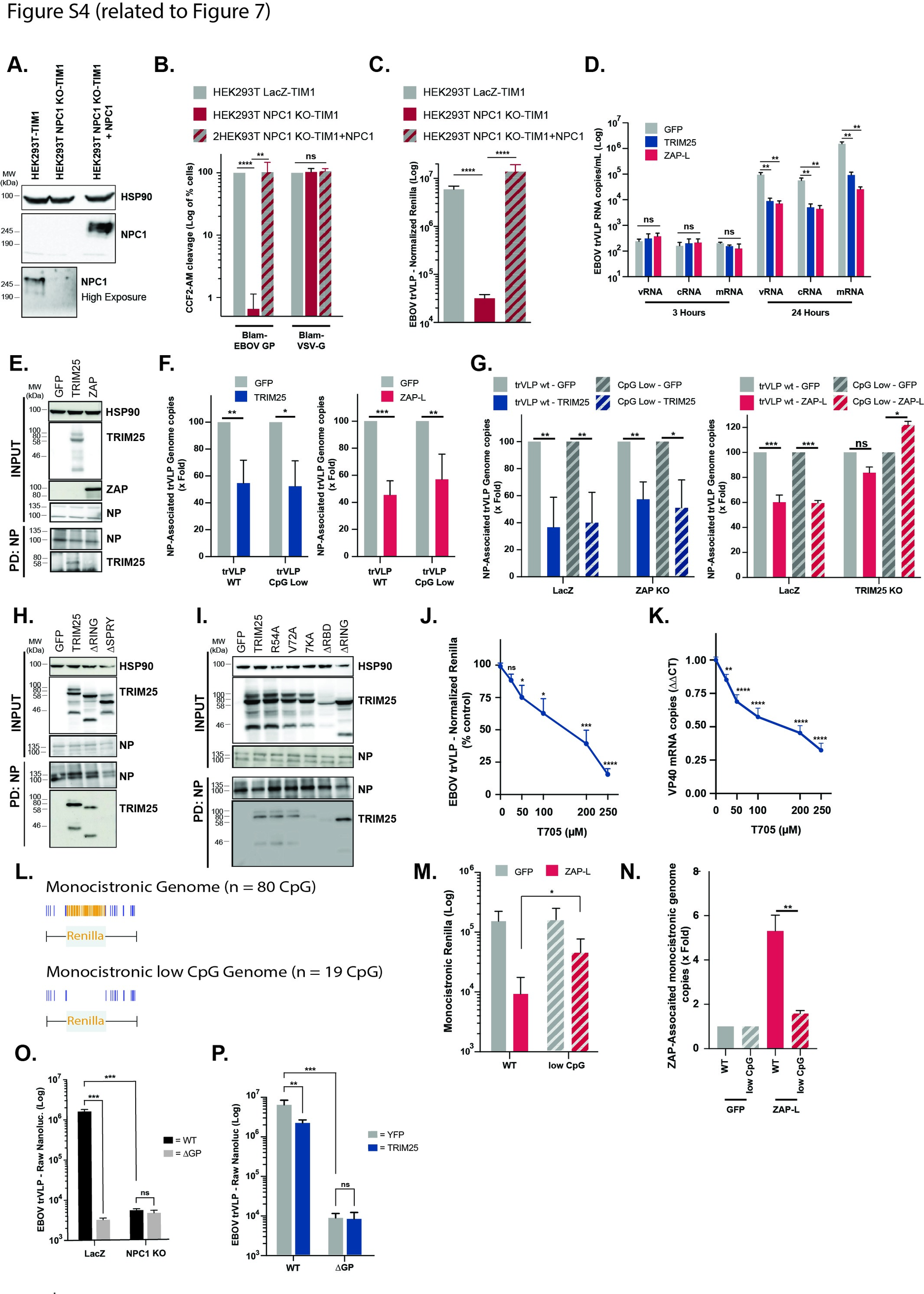

Supplement: S4 Fig — EBOV trVLP replication is dependent on the entry factor NPC1 and is sensitive to transcription inhibitor T705 (Favipiravir). (A) Cellular lysates from HEK293T LacZ CRISPR-TIM1, HEK293T NPC1 CRISPR KO-TIM1 and HEK293T NPC1 CRISPR KO-TIM1 cells with restored expression of NPC1 (+NPC1) were analysed by western blot for HSP90 and NPC1. (B) The cells lines used in (A) were transduced either with BlaVP40-EBOV-GP or BlaVP40-VSV-G virus-like particles, and 24 hours later the percentages of cells presenting cleavage of CCF2-AM dye were determined by flow cytometry as readout for viral particle entry. (C) EBOV trVLP normalized reporter activity on the upper mentioned cell lines (p1) transfected with EBOV RNP proteins and infected the following day with a fixed amount of EBOV trVLPs. Reporter activities were measured 24 hours later. (D) Quantification of intracellular viral RNA levels in HEK293T-TIM1 target cells 3 and 24 hours post-infection with a fixed amount of EBOV trVLPs. Prior to infection the cells were transfected with EBOV RNP expressing plasmids together with GFP (grey), TRIM25 (blue) or ZAP-L (red). Strand-specific reverse transcription primers were used on total RNA extracted from cells to generate cDNAs for minigenomic RNA (vRNA), complementary RNA (cRNA), and mRNA, followed by RT-qPCR analysis. (E) HEK293T LacZ CRISPR-TIM1 cells were transfected with GFP, TRIM25 or ZAP-L prior to infection with EBOV trVLPs. 3 hours post-infection cells were UV cross-linked, and EBOV NP from incoming virions were immunoprecipitated from lysates with an anti-NP antibody. Cellular lysates and pull-down samples were analysed by western blot for HSP90, TRIM25, NP and ZAP. (F) Relative quantification of NP-associated RNA in HEK293T LacZ CRISPR-TIM1 cells transfected with GFP (grey), TRIM25 (blue) or ZAP-L (red) prior to infection with wild-type or CpG low EBOV trVLPs. 3 hours post-infection cells were UV cross-linked, and EBOV NP from incoming virions was immunoprecipitated from lysat [file ppat.1010530.s004.tif]
