# Supplementary material for: TRIM25 and ZAP target the Ebola virus ribonucleoprotein complex to mediate interferon-induced restriction
Source: PLoS Pathog. 2022 May 9;18(5):e1010530. doi: 10.1371/journal.ppat.1010530 (PMC9119685; doi:10.1371/journal.ppat.1010530)
Supplement: S1 Table — (PDF) [file ppat.1010530.s005.pdf]

Supplemental Table 1
Large Scale ISG Screen: EboV trVLP in HEK293T cells – p1 and p2 values (Page 1 of 4)

Gene	P2 Replication (% control)	Z score	P1 Replication (% control)	Z score	Gene	P2 Replication (% control)	Z score	P1 Replication (% control)	Z score
P2RY6	0.4	-1.24	41.5	-1.23	MCOLN	28.0	-0.89	40.4	-1.25
BST2/THN	0.5	-1.24	91.8	-0.18	ZNF313	28.1	-0.89	53.5	-0.98
CCL5	0.5	-1.24	106.0	0.11	PDGFRL	28.3	-0.89	100.0	-0.01
NMI	0.6	-1.24	0.4	-2.08	PNPT1	28.7	-0.88	103.6	0.06
APOL6	0.6	-1.24	188.5	1.83	PHF15	28.7	-0.88	119.2	0.39
ARHGAP17	1.9	-1.22	57.9	-0.88	GBP5	28.9	-0.88	116.9	0.34
WHDC1	2.3	-1.21	32.0	-1.42	JAK2	29.6	-0.87	96.8	-0.08
B2M	2.8	-1.21	108.2	0.16	ATF3	30.3	-0.86	116.4	0.33
OAS1	2.9	-1.21	76.4	-0.50	C10orf10	30.5	-0.86	41.8	-1.22
CTCFL	3.3	-1.20	55.6	-0.93	AIM2	30.5	-0.86	70.3	-0.63
DNAPTP6	3.4	-1.20	58.4	-0.88	CCL19	30.6	-0.86	78.0	-0.47
PPM1K	4.2	-1.19	35.0	-1.36	RAB27A	30.9	-0.86	58.7	-0.87
MT1G	4.3	-1.19	57.1	-0.90	IFI35	31.1	-0.85	174.6	1.54
IFIH1 / MDA5	4.3	-1.19	17.9	-1.72	FBXO6	31.4	-0.85	52.3	-1.00
FNDC4	8.1	-1.14	142.9	0.88	UNC93B1	31.9	-0.84	28.2	-1.50
C5orf39	8.3	-1.14	15.0	-1.78	HLA-E	32.1	-0.84	66.7	-0.70
SOCS2	8.5	-1.14	77.3	-0.48	GTPBP2	32.5	-0.84	184.2	1.74
FCGR1A	10.1	-1.12	148.1	0.99	GLRX	32.8	-0.83	60.9	-0.82
MCL1	11.2	-1.10	124.4	0.50	CX3CL1	33.1	-0.83	212.9	2.33
ARHGEF3	11.4	-1.10	15.9	-1.76	TAP2	33.2	-0.83	145.1	0.92
FLT1	11.7	-1.10	67.9	-0.68	XAF1	33.4	-0.82	55.0	-0.95
MAP3K14	11.9	-1.09	63.6	-0.77	WARS	33.7	-0.82	112.2	0.24
OASL	12.1	-1.09	123.2	0.47	IFNGR1	34.1	-0.82	51.7	-1.01
TNK2	12.4	-1.09	165.0	1.34	SLC25A28	34.5	-0.81	105.1	0.10
GBP2	13.2	-1.08	74.2	-0.55	VIPERIN	34.7	-0.81	98.2	-0.05
GALNT2	13.7	-1.07	133.9	0.69	ZBP1	34.9	-0.81	54.1	-0.96
FFAR2	14.9	-1.06	43.0	-1.20	MYD88	35.4	-0.80	46.4	-1.12
CCDC92	15.0	-1.06	79.8	-0.43	CASP7	35.5	-0.80	71.3	-0.61
LMO2	15.4	-1.05	80.7	-0.41	NDC80	35.6	-0.80	66.9	-0.70
SLC15A3	16.0	-1.04	174.0	1.53	MAFF	36.0	-0.79	97.5	-0.06
HERC5	16.6	-1.04	77.1	-0.49	APOBEC3A	36.5	-0.79	56.2	-0.92
TXNIP	16.6	-1.03	80.5	-0.42	ZNF385B	36.6	-0.78	61.2	-0.82
UBA7	17.5	-1.02	55.8	-0.93	DEFB1	37.1	-0.78	80.9	-0.41
PIM3	17.5	-1.02	87.6	-0.27	XRN	37.1	-0.78	88.0	-0.26
TRIM25	17.7	-1.02	25.4	-1.56	SAMD4A	38.3	-0.76	62.5	-0.79
TRIM56	19.2	-1.00	25.6	-1.56	SMAD3	38.7	-0.76	80.5	-0.42
SLFN12	19.4	-1.00	56.1	-0.92	STAT2	39.9	-0.74	105.0	0.09
STEAP4	19.8	-1.00	86.3	-0.30	PUS1	39.9	-0.74	120.3	0.41
CXCL10	19.8	-0.99	90.5	-0.21	IFIT3	40.9	-0.73	92.5	-0.17
MAB21L2	19.9	-0.99	130.6	0.62	C15orf48	41.2	-0.73	63.9	-0.76
FAM46C	20.6	-0.99	38.6	-1.29	IFITM1	41.3	-0.73	109.3	0.18
MX2	21.6	-0.97	49.9	-1.05	GK	41.4	-0.72	67.8	-0.68
GBP1	21.8	-0.97	78.6	-0.46	SAT1	42.4	-0.71	93.9	-0.14
PABPC4	22.0	-0.97	20.4	-1.67	KIAA0082	42.5	-0.71	50.9	-1.03
SERPINE1	22.3	-0.96	52.2	-1.00	TNFRSF10A	43.7	-0.70	177.3	1.59
SERPING1	23.2	-0.95	68.0	-0.68	TNFAIP3	44.3	-0.69	182.0	1.69
C5orf27	23.5	-0.95	81.9	-0.39	IL28RA	44.4	-0.69	80.4	-0.42
SERPINB9	23.8	-0.94	155.3	1.14	NFIL3	45.0	-0.68	78.1	-0.47
IRF1	24.0	-0.94	33.2	-1.40	RARRES3	45.1	-0.68	97.0	-0.07
HCP5	24.5	-0.94	110.4	0.21	BIRC3	46.4	-0.66	67.0	-0.70
ODC1	24.6	-0.93	151.4	1.06	HEG1	46.5	-0.66	74.1	-0.55
TRIM14	24.7	-0.93	65.9	-0.72	C9ofr19	46.5	-0.66	85.1	-0.32
CLEC4D	25.3	-0.93	68.9	-0.66	HSPA6	46.6	-0.66	124.7	0.50
IFIT1	25.4	-0.92	96.3	-0.09	USP18	47.4	-0.65	52.6	-1.00
CNP	25.5	-0.92	156.1	1.15	DDX60	47.7	-0.65	87.9	-0.26
NCF1	26.8	-0.91	92.8	-0.16	EPAS1	48.0	-0.64	193.5	1.93
CD274	26.9	-0.91	88.2	-0.26	SAMHD1	48.2	-0.64	55.7	-0.93
IFI27	27.5	-0.90	95.5	-0.10	FAM125B	48.4	-0.64	60.8	-0.82
CEACAM1	27.6	-0.90	59.7	-0.85	STAP1	49.1	-0.63	50.5	-1.04

Large Scale ISG Screen: EboV trVLP in HEK293T cells – p1 and p2 values (Page 2 of 4)

Gene	P2 Replication (% control)	Z score	P1 Replication (% control)	Z score	Gene	P2 Replication (% control)	Z score	P1 Replication (% control)	Z score
DYNLT1	49.5	-0.62	77.0	-0.49	ISG20	68.1	-0.39	44.7	-1.16
SSBP3	49.5	-0.62	51.2	-1.03	TRAFD1	68.1	-0.39	126.7	0.54
RBM43	49.7	-0.62	75.9	-0.51	MX1	68.2	-0.39	72.0	-0.59
AKT3	49.8	-0.62	109.0	0.18	HLA-F	68.4	-0.39	125.4	0.52
ANGPTL1	50.5	-0.61	97.3	-0.07	TAP1	68.6	-0.38	98.8	-0.04
FAM70A	50.7	-0.61	75.8	-0.51	LGALS3	69.9	-0.37	85.9	-0.30
UPP2	50.7	-0.61	50.0	-1.05	HLA-C	70.2	-0.36	123.5	0.48
SIRPA	51.0	-0.60	38.2	-1.29	CASP1	70.4	-0.36	43.4	-1.19
CCND3	52.1	-0.59	163.9	1.32	RPL22	70.8	-0.35	116.2	0.33
RNF19B	52.2	-0.59	82.5	-0.37	APOL3	71.2	-0.35	75.3	-0.52
MTHFD2L	52.5	-0.59	73.4	-0.56	PSCD1	72.4	-0.34	67.5	-0.69
EIF3EIP	53.2	-0.58	147.1	0.97	SCO2	72.6	-0.33	166.5	1.37
THBD	53.3	-0.57	201.1	2.09	TRIM38	73.4	-0.32	116.5	0.33
NT5C3	53.5	-0.57	84.5	-0.33	IFI44	75.2	-0.30	79.5	-0.44
PSMB8	53.6	-0.57	80.4	-0.42	IFI44L	75.7	-0.29	30.0	-1.47
MS4A4A	54.0	-0.57	52.2	-1.00	AXUD1	75.9	-0.29	90.9	-0.20
SECTM1	54.0	-0.57	45.8	-1.14	MAFF	76.4	-0.28	102.4	0.04
ABLIM3	54.5	-0.56	53.7	-0.97	ARNTL	76.6	-0.28	154.5	1.12
FAM46A	54.9	-0.55	52.3	-1.00	TLR3	76.9	-0.28	106.4	0.12
RTP4	55.1	-0.55	87.1	-0.28	CXCL9	77.0	-0.28	85.2	-0.32
CMAH	55.2	-0.55	115.0	0.30	C9orf91	77.0	-0.28	120.6	0.42
MT1M	55.9	-0.54	148.2	0.99	IFI16	77.7	-0.27	94.9	-0.12
SOCS1	56.6	-0.53	108.5	0.17	PSMB9	77.7	-0.27	126.0	0.53
DDX58	57.1	-0.53	33.2	-1.40	IFITM3	78.3	-0.26	64.8	-0.74
BAG1	57.2	-0.53	122.4	0.45	ALDH1A1	78.9	-0.25	69.4	-0.65
UNC84B	57.3	-0.52	96.8	-0.08	ERLIN1	79.2	-0.25	72.9	-0.57
C19orf66	57.4	-0.52	96.9	-0.08	CCL8	79.3	-0.25	51.3	-1.02
B4GALT5	57.9	-0.52	104.5	0.08	INDO	79.8	-0.24	88.7	-0.25
SPTLC2	58.1	-0.51	88.3	-0.25	RNASE4	80.1	-0.24	161.0	1.26
BUB1	58.6	-0.51	74.2	-0.55	AGPAT9	80.2	-0.24	69.0	-0.66
MOV10	59.1	-0.50	513.4	8.58	DCP1A	82.3	-0.21	108.3	0.16
SAMD9L	59.4	-0.50	66.9	-0.70	HERC6	82.5	-0.21	124.0	0.49
TMEM51	60.0	-0.49	104.9	0.09	IL17RB	82.9	-0.20	152.4	1.08
VAMP5	60.2	-0.49	65.5	-0.73	JUNB	84.0	-0.19	115.9	0.32
BLZF1	60.2	-0.49	109.1	0.18	N4BP1	84.5	-0.18	59.3	-0.86
PRKD2	60.4	-0.49	150.6	1.04	EPSTI1	84.6	-0.18	90.3	-0.21
CHMP5	60.6	-0.48	126.8	0.55	TGFB1	85.1	-0.18	126.1	0.53
DDX3X	60.6	-0.48	59.7	-0.85	SAA1	85.1	-0.18	204.6	2.16
TMEM49	60.9	-0.48	118.1	0.36	PFKFB3	85.6	-0.17	137.3	0.76
SP110	61.2	-0.48	131.9	0.65	C2orf31	85.9	-0.17	125.1	0.51
CCNA1	62.6	-0.46	128.5	0.58	PCTK3	86.5	-0.16	113.5	0.27
IRF9	62.9	-0.46	90.5	-0.21	PARP12	86.7	-0.16	103.0	0.05
PARP10	63.6	-0.45	98.6	-0.04	STARD5	87.4	-0.15	115.0	0.30
SLC1A1	63.7	-0.45	51.9	-1.01	FER1L3	87.5	-0.15	69.5	-0.65
ANKRD22	64.0	-0.44	110.6	0.21	ISG15	87.6	-0.14	135.4	0.72
C4orf32	64.0	-0.44	91.9	-0.18	MT1L	87.7	-0.14	84.7	-0.33
BCL3	64.0	-0.44	128.9	0.59	IFI6	87.9	-0.14	47.4	-1.10
MAFF	64.1	-0.44	64.8	-0.74	IFITM2	88.1	-0.14	86.1	-0.30
C4orf33	64.4	-0.44	76.6	-0.50	PMAIP1	88.5	-0.13	61.8	-0.81
C1S	64.5	-0.43	50.3	-1.04	NOS2A	89.7	-0.12	76.5	-0.50
APOL2	64.6	-0.43	87.8	-0.26	PLEKHA4	90.0	-0.11	83.1	-0.36
TRIM21	65.5	-0.42	56.4	-0.92	STAT3	90.7	-0.11	61.9	-0.80
HESX1	65.9	-0.42	97.7	-0.06	EIF2AK2	91.0	-0.10	67.4	-0.69
SNN	66.7	-0.41	57.6	-0.89	ELF1	91.5	-0.10	32.8	-1.41
LIPA	66.7	-0.41	107.0	0.13	PBEF1	92.3	-0.09	127.7	0.56
LAMP3	67.1	-0.40	107.0	0.13	RIPK2	92.6	-0.08	28.7	-1.49
CCDC109B	67.2	-0.40	51.7	-1.02	DUSP5	93.1	-0.08	142.8	0.88
OPTN	67.8	-0.39	77.9	-0.47	CSDA	93.8	-0.07	164.4	1.33
DDIT4	68.0	-0.39	130.1	0.61	OGFR	94.1	-0.06	106.3	0.12

Large Scale ISG Screen: EboV trVLP in HEK293 cells – p1 and p2 values (Page 3 of 4)

Gene	P2 Replication (% control)	Z score	P1 Replication (% control)	Z score	Gene	P2 Replication (% control)	Z score	P1 Replication (% control)	Z score
**EXT1**	94.2	-0.06	99.2	-0.03	**SLC25A30**	128.3	0.37	157.1	1.18
**RBM25**	95.1	-0.05	77.9	-0.47	**S100A8**	128.4	0.37	96.7	-0.08
**BCL2L14**	95.1	-0.05	60.0	-0.84	**SP100**	129.2	0.38	82.8	-0.37
**PRAME**	95.9	-0.04	177.4	1.60	**CRY1**	130.9	0.40	115.4	0.31
**GEM**	95.9	-0.04	80.8	-0.41	**ATP10D**	131.8	0.41	102.7	0.04
**BTN3A3**	96.1	-0.04	106.2	0.12	**IL1RN**	132.0	0.41	175.7	1.56
**TBX3**	96.4	-0.03	168.5	1.41	**IFI30**	132.5	0.42	107.7	0.15
**MICB**	98.2	-0.01	63.5	-0.77	**APOBEC3G**	132.8	0.42	68.1	-0.67
**GPX2**	98.9	0.00	114.8	0.30	**TDRD7**	132.9	0.42	123.3	0.47
**CD74**	100.2	0.01	130.0	0.61	**PTMA**	133.0	0.42	99.1	-0.03
**STAT1**	101.6	0.03	115.6	0.31	**LINCR**	134.5	0.44	83.8	-0.35
**RNF24**	102.2	0.04	91.1	-0.20	**MT1F**	134.6	0.44	49.9	-1.05
**GZMB**	102.2	0.04	75.8	-0.51	**GCA**	134.7	0.45	98.5	-0.04
**CYP1B1**	102.4	0.04	202.7	2.12	**CD163**	136.0	0.46	174.2	1.53
**LEPR**	102.4	0.04	116.0	0.32	**CPT1A**	136.6	0.47	161.5	1.27
**ADAMDEC1**	103.3	0.05	101.6	0.02	**TCF7L2**	136.7	0.47	138.7	0.79
**NPAS2**	103.6	0.06	133.9	0.69	**SLC16A1**	137.5	0.48	46.8	-1.12
**ADFP**	104.0	0.06	103.1	0.05	**PMM2**	139.5	0.51	103.8	0.07
**ANKFY1**	104.0	0.06	92.6	-0.17	**MAFB**	139.6	0.51	214.0	2.36
**ABTB2**	104.7	0.07	144.1	0.90	**THOC4**	140.0	0.51	105.6	0.10
**CD69**	105.2	0.08	108.9	0.17	**HES4**	140.8	0.52	149.2	1.01
**ZNF295**	106.3	0.09	115.1	0.30	**TIMP1**	143.6	0.56	76.6	-0.50
**AQP9**	106.6	0.09	51.1	-1.03	**IRF7**	143.7	0.56	186.1	1.78
**NCOA3**	107.0	0.10	104.8	0.09	**TAGAP**	143.7	0.56	153.9	1.11
**CCDC75**	107.9	0.11	88.5	-0.25	**LGMN**	145.4	0.58	113.9	0.28
**RGS1**	108.4	0.12	183.2	1.72	**PADI2**	146.2	0.59	278.6	3.70
**FKBP5**	108.8	0.12	78.9	-0.45	**BLVRA**	146.5	0.59	86.8	-0.29
**FLJ39739**	108.8	0.12	104.5	0.08	**SQLE**	146.9	0.60	118.5	0.37
**CXCL11**	109.6	0.13	85.0	-0.32	**NOD2**	147.4	0.61	169.5	1.43
**CLEC2B**	109.7	0.13	142.8	0.88	**GBP3**	148.4	0.62	145.5	0.93
**PXK**	110.1	0.14	68.8	-0.66	**MKX**	148.7	0.62	69.7	-0.64
**CCL2**	111.2	0.15	43.0	-1.19	**COMMD3**	149.6	0.63	64.9	-0.74
**IRF2**	111.4	0.15	145.1	0.93	**BATF2**	150.7	0.65	109.8	0.19
**TLK2**	112.3	0.16	92.1	-0.17	**DHX58**	153.6	0.68	100.6	0.00
**APOL1**	112.5	0.17	70.4	-0.63	**GCH1**	154.0	0.69	57.1	-0.90
**GAK**	112.8	0.17	121.8	0.44	**CDKN1A**	154.2	0.69	153.5	1.10
**PCTK2**	113.3	0.18	95.6	-0.10	**ENPP1**	154.9	0.70	106.5	0.12
**ZNF107**	113.5	0.18	103.5	0.06	**IL1R**	155.1	0.70	46.7	-1.12
**MARCK**	114.2	0.19	276.4	3.65	**UBE2L6**	155.3	0.70	86.3	-0.29
**CEBPD**	115.1	0.20	116.2	0.33	**PFKFB3**	155.7	0.71	275.7	3.64
**TYMP**	115.7	0.21	96.6	-0.08	**RBCK1**	158.7	0.75	140.3	0.83
**CES1**	115.9	0.21	130.3	0.62	**HSH2D**	158.8	0.75	178.5	1.62
**KIAA1618**	116.4	0.22	134.5	0.70	**RIPK2**	159.8	0.76	79.2	-0.44
**TREX1**	116.4	0.22	99.7	-0.02	**NAPA**	160.1	0.76	75.3	-0.53
**G6PC**	116.9	0.22	133.4	0.68	**CFB**	161.7	0.78	107.0	0.13
**IMPA2**	117.4	0.23	97.7	-0.06	**CLEC4A**	162.0	0.79	109.9	0.19
**DDX60**	117.5	0.23	78.6	-0.46	**IFIT5**	162.7	0.80	106.0	0.11
**C22orf28**	119.4	0.25	98.5	-0.04	**FNDC3B**	163.2	0.80	103.6	0.06
**ABCA9**	120.2	0.26	154.0	1.11	**KIAA0082**	164.0	0.81	64.6	-0.75
**MT1H**	120.9	0.27	153.7	1.10	**CD38**	167.2	0.85	77.4	-0.48
**SPSB1**	121.0	0.27	120.4	0.41	**HK2**	167.7	0.86	149.8	1.02
**SCARB2**	121.0	0.27	57.4	-0.90	**ETV6**	168.2	0.87	97.4	-0.07
**PPM1K**	122.1	0.29	63.1	-0.78	**LY6E**	168.4	0.87	66.6	-0.71
**MAP3K5**	122.7	0.29	5.3	-1.98	**TFEC**	170.4	0.89	98.1	-0.05
**CRP**	124.0	0.31	98.4	-0.04	**SLFN5**	170.5	0.89	64.6	-0.75
**IRF2**	124.9	0.32	59.8	-0.85	**LAP3**	172.5	0.92	110.1	0.20
**RGL-1**	125.8	0.33	153.1	1.09	**NRN1**	172.7	0.92	91.3	-0.19
**GMPR**	126.4	0.34	93.6	-0.14	**ZNF107**	174.4	0.94	86.4	-0.29
**CCL4**	127.4	0.35	72.7	-0.58	**PDK1**	174.5	0.94	94.9	-0.12

Large Scale ISG Screen: EboV trVLP in HEK293 cells – p1 and p2 values

Gene	P2 Replication (% control)	Z score	P1 Replication (% control)	Z score	Gene	P2 Replication (% control)	Z score	P1 Replication (% control)	Z score
LGALS9	175.7	0.96	166.6	1.37	LRG1	246.4	1.85	132.9	0.67
IL6ST	176.3	0.97	106.9	0.13	TLR7	248.7	1.88	63.9	-0.76
RASGEF1B	179.1	1.00	72.8	-0.58	ADAR	250.4	1.90	180.5	1.66
RASSF4	183.0	1.05	118.3	0.37	TNFSF10	255.5	1.96	99.2	-0.03
DTX3L	188.2	1.12	110.6	0.21	CD80	262.4	2.05	63.1	-0.78
CCR1	189.7	1.13	70.7	-0.62	ARG2	269.5	2.14	132.9	0.67
CREB3L3	192.2	1.17	152.9	1.09	TRIM34	271.6	2.16	103.1	0.05
LOC400759	193.2	1.18	85.1	-0.32	HPSE	273.0	2.18	109.3	0.18
IL15	193.6	1.18	55.3	-0.94	ZC3HAV1	273.6	2.19	70.7	-0.62
NUP50	196.4	1.22	87.3	-0.27	PML	277.0	2.23	267.3	3.46
IGFBP2	197.3	1.23	141.9	0.86	PI4K2B	278.1	2.24	99.9	-0.01
GJA4	198.1	1.24	104.7	0.09	MASTL	280.4	2.27	161.7	1.27
HLA-G	198.5	1.25	82.1	-0.38	FLJ23556	291.6	2.41	99.0	-0.03
FUT4	199.1	1.25	234.5	2.78	PAK3	295.7	2.46	59.2	-0.86
PNRC1	200.1	1.27	80.6	-0.41	TNFSF13B	306.0	2.59	90.3	-0.21
LAMP3	206.0	1.34	143.4	0.89	ULK4	312.9	2.68	90.2	-0.21
EHD4	206.6	1.35	77.7	-0.47	MSR1	317.5	2.74	124.4	0.50
SIGLEC1	206.9	1.35	177.7	1.60	TNFAIP6	323.0	2.81	127.8	0.57
C6orf150	213.1	1.43	142.6	0.87	MAX	331.3	2.91	146.1	0.95
FAM134B	217.7	1.49	94.5	-0.12	KIAA0040	345.5	3.09	121.8	0.44
PLSCR1	221.2	1.53	63.1	-0.78	OAS3	371.0	3.41	148.0	0.99
GBP4	223.4	1.56	92.3	-0.17	IFIT2	406.4	3.85	103.0	0.05
ADM	223.6	1.56	124.9	0.51	AHNAK2	408.6	3.88	125.6	0.52
VEGFC	224.7	1.57	134.0	0.69	TRIM5	410.9	3.91	80.4	-0.42
ETV7	225.2	1.58	80.0	-0.43	OAS2	433.0	4.19	104.0	0.07
AMPH	228.5	1.62	190.9	1.88	CD9	491.6	4.92	188.2	1.82
IL15RA	229.8	1.64	135.0	0.72					
