# Supplementary material for: TRIM25 and ZAP target the Ebola virus ribonucleoprotein complex to mediate interferon-induced restriction
Source: PLoS Pathog. 2022 May 9;18(5):e1010530. doi: 10.1371/journal.ppat.1010530 (PMC9119685; doi:10.1371/journal.ppat.1010530)
Supplement: S2 Table — (DOCX) [file ppat.1010530.s006.docx]

| **Table S2 – CRISPR Guides** | |
| --- | --- |
| LacZ Guide | 5’- CGA TTA AGT TGG GTA ACG CC -3’ |
| TRIM25 CRISPR Guide | 5’- GAG CCG GTC ACC ACT CCG TG -3’ |
| ZAP CRISPR Guide | 5’- ACT TCC ATC TGC CTT ACC GG -3’ |
| RIG-I CRISPR Guide | 5’- GGG TCT TCC GGA TAT AAT CC -3’ |
| MAVS CRISPR Guide | 5’- ATT GCG GCA GAT ATA CTT AT -3’ |
| MAVS & miniMAVS CRISPR Guide 7 | 5’- TCT GGG GCT GAG CGT CTG CA -3’ |
| TBK1 CRISPR Guide | 5’- AGA GCA CTT CTA ATC ATC TG -3’ |
| NPC1 CRISPR Guides | 5’- GCT CAC AAA ACA GGT TCA GT -3’  5’- ACA GTC GTC TTG CTG TCG AG –3’ |
| KHNYN CRISPR Guides | 5’-GGG GGT GAG CGT CCT TCC GA-3’ |
