# Supplementary material for: TRIM25 and ZAP target the Ebola virus ribonucleoprotein complex to mediate interferon-induced restriction
Source: PLoS Pathog. 2022 May 9;18(5):e1010530. doi: 10.1371/journal.ppat.1010530 (PMC9119685; doi:10.1371/journal.ppat.1010530)
Supplement: S3 Table — (DOCX) [file ppat.1010530.s007.docx]

| **Table S3 – Primers used for cloning** | |
| --- | --- |
| EcoRI_Kz_TRIM25wt_Fwd | GCGCGAATTCGCCACCATGGCAGAGCTGTGCCCCCTGG |
| TRIM25 ∆RING_Fwd EcoRI | GCGCGAATTCGCCACCATGCGCGCCGTCTACCAGGCGCGACCGC |
| TRIM25 ∆SPRY_Rev XhoI | CGCGCGCTCGAGAATTTTAATGTAATACTCCAGGAGCTC |
| TRIM25wt_Rev_XhoI | CGCGCGCTCGAGCTACTTGGGGGAGCAGATGG |
| TRIM25wt_CR_Fwd | GAGCCGGTGACAACACCTTGCGGCCACAA |
| TRIM25wt_CR_Rev | TTGTGGCCGCAAGGTGTTGTCACCGGCTC |
| TRIM25_CR_L7A_Fwd | GCGCGAATTCGCCACCATGGCAGAGCTGTGCCCCGCGGCCGAGGAGCT |
| TRIM25_CR_L17/18R_Fwd | GCTGTCGTGCTCCATCTGCCGGCGACCCTTCAAGGAGCCGGTC |
| TRIM25_CR_L17/18R_Rev | GACCGGCTCCTTGAAGGGTCGCCGGCAGATGGAGCACGACAGC |
| TRIM25_CR_Q52R_Fwd | CGCCATACCTGTGCCCGCGATGCCGCGCCGTCTACCAGG |
| TRIM25_CR_Q52R_Rev | CCTGGTAGACGGCGCGGCATCGCGGGCACAGGTATGGCG |
| TRIM25_CR_R54A_Fwd | CCATACCTGTGCCCGCAGTGCGCGGCCGTCTACCAGGCGCG |
| TRIM25_CR_R54A_Rev | CGCGCCTGGTAGACGGCCGCGCACTGCGGGCACAGGTATGG |
| TRIM25_CR_K65A_Fwd | GGCGCGACCGCAGCTGCACGCGAACACGGTGCTGTGCAACG |
| TRIM25_CR_K65A_Rev | CGTTGCACAGCACCGTGTTCGCGTGCAGCTGCGGTCGCGCC |
| TRIM25_CR_N66A_Fwd | CCGCAGCTGCACAAGGCCACGGTGCTGTGCAAC |
| TRIM25_CR_N66A_Rev | GTTGCACAGCACCGTGGCCTTGTGCAGCTGCGG |
| TRIM25_CR_V68A_Fwd | AGCTGCACAAGAACACGGCGCTGTGCAACGTGGTGG |
| TRIM25_CR_V68A_Rev | CCACCACGTTGCACAGCGCCGTGTTCTTGTGCAGCT |
| TRIM25_CR_L69A_Fwd | CTGCACAAGAACACGGTGGCGTGCAACGTGGTGGAGC |
| TRIM25_CR_L69A_Rev | GCTCCACCACGTTGCACGCCACCGTGTTCTTGTGCAG |
| TRIM25_CR_N71D_Fwd | AACACGGTGCTGTGCGACGTGGTGGAGCAGTTCCTG |
| TRIM25_CR_N71D_Rev | CAGGAACTGCTCCACCACGTCGCACAGCACCGTGTT |
| TRIM25_CR_V72A_Fwd | ACGGTGCTGTGCAACGCGGTGGAGCAGTTCCTGCA |
| TRIM25_CR_V72A_Rev | TGCAGGAACTGCTCCACCGCGTTGCACAGCACCGT |
| TRIM25_CR_K117R_Fwd | GAGGCCGCCGTGAGGACGTGCTTGGTGTGC |
| TRIM25_CR_K117R_Rev | GCACACCAAGCACGTCCTCACGGCGGCCTC |
| TRIM25_CR_Y245A_Fwd | GTGGAGCAGCTACAACAAGAAGCCACGGAAATGAAGGCTCTCT |
| TRIM25_CR_Y245A_Rev | AGAGAGCCTTCATTTCCGTGGCTTCTTGTTGTAGCTGCTCCAC |
| TRIM25_CR_Y252A_Fwd | ACGGAAATGAAGGCTCTCGCGGACGCCTCAGAGACCAC |
| TRIM25_CR_Y252A_Rev | GTGGTCTCTGAGGCGTCCGCGAGAGCCTTCATTTCCGT |
| TRIM25_CR_RHK_AAA Fwd | CGACCTGGAGGCCACCCTGGCCGCAGCCCTAACTGTCATGTACAGTCAGATC |
| TRIM25_CR_RHK_AAA Rev | GATCTGACTGTACATGACAGTTAGGGCTGCGGCCAGGGTGGCCTCCAGGTCG |
| TRIM25_CR_∆RBD_Fwd | ACCGCCCACAACAAAAAGGGGATCCACTACTGGGAGGTGGAG |
| TRIM25_CR_∆RBD_Fwd | GTAGTGGATCCCCTTTTTGTTGTGGGCGGTGTTGTAGTCCAG |
| TRIM25_CR_7KA_Fwd 1 | CGCAGAGGAAGCGGCATCCGCGGCACCTCCCCCTGTCCCTGCCTTA |
| TRIM25_CR_7KA_Fwd 2 | ACACGCCCTGTGGCGGCGGTCTCCGCAGAGGAAGCGGCATCC |
| TRIM25_CR_7KA_Rev 1 | TGCCGCTTCCTCTGCGGAGACCGCCGCCACAGGGCGTGTGGATTTGTG |
| TRIM25_CR_7KA_Rev 2 | AGGGACAGGGGGAGGTGCCGCGGATGCCGCTTCCTCTGCGGAG |
| MAVS Not I Fwd | AAGCGCGGCCGCGCAGCAATGCCGTTTGCTGAAGACAAG |
| MAVS CR Fwd | ATTCAGAGCAAGCACTGCAGACGCTCAGCC |
| MAVS CR Rev | GGCTGAGCGTCTGCAGTGCTTGCTCTGAAT |
| MAVS M1A NotI Fwd | AGGCGCGGCCGCGCAGCAGCGCCGTTTGCTGAAGACAAG |
| MAVS M142A Fwd | AGGAGCCAAGTTACCCCGCGCCTGTCCAGGAGACCCAG |
| MAVS M142A Rev | CTGGGTCTCCTGGACAGGCGCGGGGTAACTTGGCTCCT |
| MAVS Rev XhoI | TGCTCTCGAGCTAGTGCAGACGCCGCC |
| XhoI_TIM1_Fwd | AGACCTCGAGATCCCATAATGCATCTTCAAGTGGTCATCTTAAGC |
| TIM1_Rev_NotI | ATGCGCGGCCGCTCATGGGCGTAAACTCTCAAAGAGCAC |
| EcoRI_NPC1_Fwd | GAATTCGCCACCATGACCGCTCGCGGCCTGGCCC |
| NPC1_Rev_XhoI | CGCGCTCGAGGGGGGCCTCCACATCCCGGCAGGC |
| P4 cis vRNA BlpI F | GACCCGTTTAGAGGCCCCAA |
| P4 cis low CpG TRAIL F | GGGTGCTGAAGAATGAGCAGTAAATTACCGTGCATAGTATCCTGATACTT |
| P4 cis low CpG TRAIL R | AAGTATCAGGATACTATGCACGGTAATTTACTGCTCATTCTTCAGCACCC |
| P4 cis vRNA XmaI R | TCGATCCCGGGTTAATACGACTCA |
