# Supplementary material for: TRIM25 and ZAP target the Ebola virus ribonucleoprotein complex to mediate interferon-induced restriction
Source: PLoS Pathog. 2022 May 9;18(5):e1010530. doi: 10.1371/journal.ppat.1010530 (PMC9119685; doi:10.1371/journal.ppat.1010530)
Supplement: S4 Table — (DOCX) [file ppat.1010530.s008.docx]

| **Table S4 – Primers and probes used for cDNA synthesis and RT-qPCR** | |
| --- | --- |
| Trailer qPCR_F | 5’- CCA AAA CAC TAT TCC ATC TGA CAG GA -3’ |
| Trailer qPCR_R | 5’- TGC CGC AAT GAA TTT AAC GC -3’ |
| Trailer qPCR probe | 5’- ATG AGC CCA GAC CTT TCG TT -3’ |
| VP40 qPCR_F | 5’- AGT TGG ACT GGC GGA AGA AC -3’ |
| VP40 qPCR_R | 5’- CAG AGT CAA TCG GCT GGG TC -3’ |
| VP40 qPCR probe | 5’- AAG CCT GGT TTC CAA TTC GC -3’ |
| GAPDH Taqman Assay | Applied Biosystems (Cat# Hs99999905_m1) |
| OligodT | 5’- TTT TTT TTT TTT TTT T -3’ |
| L-Pol RNA qPCR F | 5’- CCT ACG AAC AAT GTC CGC -3’ |
| L-Pol RNA qPCR R | 5’- CTG CGG AAG GAC ATT TTG -3’ |
| L-Pol qPCR probe | 5’- TTG TTA GTG CAT GGC CGA AC -3’ |
| EBOV -vRNA RT | 5’ CCT CTC TCC CTG CGT GAT AAT C -3’ |
| EBOV +cRNA RT | 5’- GCC TTC TGA TGA GCG TGG TC -3’ |
