# Supplementary material for: TRIM25 and ZAP target the Ebola virus ribonucleoprotein complex to mediate interferon-induced restriction
Source: PLoS Pathog. 2022 May 9;18(5):e1010530. doi: 10.1371/journal.ppat.1010530 (PMC9119685; doi:10.1371/journal.ppat.1010530)
Supplement: Attachment — Submitted filename: Galao RP_Response to Reviewers_PLOS Path.docx [file ppat.1010530.s009.docx]

**Reviewer's Responses to Questions**

**Part I - Summary**
Please use this section to discuss strengths/weaknesses of study, novelty/significance, general execution and scholarship.

**Reviewer #1:** The manuscript ‘TRIM25 and ZAP target the Ebola virus ribonucleoprotein complex to mediate interferon-induced restriction’ by Galao et al. examines the potential role of interferon-stimulated genes (ISGs) in restricting the replication and transcription of Ebola virus (EBOV) and describe TRIM25 and ZAP as ISGs that interdependently antagonize the replication and transcription of EBOV transcription and replication competent virus like particles (trVLP). The manuscript first describes the sensitivity of the trVLP system to type I interferon (IFN-I), and then identifies TRIM25 as host factor that antagonizes EBOV trVLP production. Subsequent experiments examined the mechanism through which TRIM25 acts, and the authors suggest that TRIM25 ubiquitinates EBOV nucleoprotein (NP), facilitating the dissociation of NP from the viral minigenome RNA allowing ZAP to bind to CpG rich regions of the minigenome.

In general, this manuscript presents a potentially important antiviral mechanism against Ebola virus, which would be of interest to the field. Although the paper presents evidence for interaction among TRIM25, ZAP, and EBOV vRNP in the context of an interferon-stimulated cell, the functional significance is lacking especially when considering the strong IFN-I inhibition during EBOV infection as well as the use of a surrogate VLP system. Although it is understandable the need of establishing model systems to study Ebola in lower biocontainment, this unfortunately diminishes the relevance of the study. There are also some inconsistencies, and controls for some experiments were not very carefully performed. Overall, the authors show a connection between ISGs ZAP and TRIM25 that could represent an important anti-EBOV mechanism but the function in the context of an IFN-I and EBOV replication is unclear.

We thank the reviewer for the thorough revision of our manuscript and the helpful criticism. We agree that the impact of this work would be higher if the experiments were not limited to using the Ebola virus trVLP system as a surrogate but, unfortunately, we were not able to perform experiments with full-length Ebola virus due to biocontainment and pandemic-related constraints in a timely manner. However, we hope to have both conveniently addressed the reviewer’s comments in our response below, and that our revised manuscript is now clearer in its methodology and results, and thus better supporting our interpretation/findings/conclusion that TRIM25 and ZAP are relevant antiviral effectors against EBOV replication.

**Reviewer #2:** In their manuscript entitled “TRIM25 and ZAP target the Ebola virus ribonucleoprotein complex to mediate interferon-induced restriction,” Galao and colleagues present evidence for a novel EBOV-specific restriction pathway mediated by the cellular proteins TRIM25 and ZAP. The authors use the EBOV transcription and replication competent virus like particle (trVLP) system to screen for IFN-induced restriction of trVLP production. From this screen the authors identify TRIM25 as a restriction factor, and they subsequently show that its activity is independent of RIG-I but dependent on MAVS. The authors further demonstrate that ZAP is necessary for the TRIM25-mediated restriction of EBOV trVLPS and that, together, these two proteins promote the ubiquitination of NP and its dissociation from the trVLP genome. The conclusions of this paper are interesting, and they may have important implications on the EBOV replication cycle. The authors should be commended for their thorough investigation and the significant amount of work depicted herein. Likewise, the manuscript is well organized and well written. Nevertheless, there are a few aspects of the manuscript that could be improved, as described below.

We thank the reviewer for the supportive comments on our study, and the helpful suggestions its improvement. We hope to have properly addressed the points raised by this reviewer in our response below and in the revised version of our manuscript.

**Reviewer #3:** In this manuscript, the authors identified TRIM25 as a potent EBOV inhibitory host factor by use of EBOV trVLP-based interferon-stimulated genes screen. They demonstrated that TRIM25 and a related adaptor molecule ZAP contribute to type I IFN-mediated inhibition of replication process of trVLP. They also confirmed that TRIM25 interacts with the EBOV NP and promotes its ubiquitination, leading to dissociation of NP from the vRNAs. The authors further observed that ZAP targets the CpG-rich region of vRNA. Taken together, the authors concluded that TRIM25 facilitates interaction of vRNAs with ZAP by exposing CpG-rich vRNA region, resulting in suppression of trVLP replication. Although their findings suggest the potential contribution of TRIM25 in host defense against EBOV infection, there are several fundamental problems in the experimental design. Firstly, it is not clear why the authors selected a glioma cell line for evaluation of TRIM25 functions in trVLP system. Some critical experiments were not conducted appropriately. Moreover, the significance of TRIM25 and related adaptor molecules in the inhibition of trVLP replication was not robust. Please refer to my specific comments as follows:

We thank the reviewer for taking the time to critically evaluate our manuscript and providing helpful comments and suggestions to improve the study. We took into consideration the points raised and hope that the revised version of the manuscript is now clearer and conveniently addresses the important aspects highlighted. We have responded below to both general and specific comments.

**Part II – Major Issues: Key Experiments Required for Acceptance**
Please use this section to detail the key new experiments or modifications of existing experiments that should be absolutely required to validate study conclusions.

Generally, there should be no more than 3 such required experiments or major modifications for a "Major Revision" recommendation. If more than 3 experiments are necessary to validate the study conclusions, then you are encouraged to recommend "Reject".

**General points:**

**Reviewer #1:**

1) A main concern for this paper is that the roles of TRIM25 are only observed following pre-treatment with type I IFN. The authors claim while describing figure 2I that “in the absence of IFN treatment, TRIM25 knockout HEK293T cells gave significantly higher Rluc activity in p0 producer cells and yielded significant higher levels of virus (p1 infectivity)”, yet in Figures 2F and 2G there is no difference between WT and TRIM25 knockout cells in the absence of IFN-I. Also, the authors first identified TRIM25 through an over-expression screening that screened in the context of IFN-I treated cells. Since the phenotype is observed only in the presence of IFN, alternative possibility to the model described by the authors is that TRIM25 somehow affects the IFN signalling pathway (downstream of the IFN receptor), leading to effects on other ISGs that equally could affect EBOV replication. This is possibility is not ruled out. In addition, the authors should discuss in the manuscript when and how during infection the role for TRIM25 they are describing may be relevant. The levels of IFN-I for these studies to see a strong phenotype is also very high relative to physiological levels of IFN-I that are produced in response to a viral infection. Overall, the reliance on pre-stimulation with IFN-I or TRIM25 over-expression to see a phenotype bring into question whether the findings reflect the phenotype one would observe during EBOV infection.

This first general point addresses several issues that will either be replied to here and/or redirected to specific comments below. For those aspects of experimental setups that might have not been clearly described in the original version of the manuscript, we have now tried to properly address them throughout the text:

- In a couple of sentences, the reviewer mentions that the roles of TRIM25, and the ISG-screen itself, were only observed after a pre-treatment with IFN-I. This interpretation is not correct and may stem from a misreading of the diagram on the old Figure S1A (now Figure 1A). The over-expression screen, as well as all the experiments involving the transfection of TRIM25 and ZAP, **were performed in the absence of any treatment with IFN-I**. The goal of the screen was to identify ISGs that specifically have an antiviral effect against EBOV and pre-treating the cells with IFN-I would have an enormous confounding effect in the context of an over-expression screen. It would only be required to do a pre-treatment with IFN-I if we were performing an ISG knock-down screen (siRNA/CRISPR), which is not the case. As mentioned earlier, the same applies for experiments involving over-expression of TRIM25. We tried to make this fact clearer throughout the text and figure 1A.
- Another comment is related with the fact that TRIM25 might affect the IFN pathway downstream of the IFN receptor, leading to the expression of ISGs. We believe part of this argument was based in the misconception, as we addressed in the earlier point, that all TRIM25 experiments were performed in the presence of IFN*.* However, this is a valid point and we have tried to minimise it by using the HEK293T cells in the transfection experiments. As explained in more detail in the reply to reviewer 3 point 1 and minor issue 2, we know that transfection of some ISGs might itself trigger different signalling pathways that lead to the expression of IFN-I and downstream events. And we have specifically ruled this out in the case of TRIM25 by demonstrating a non-concordance between the ability of TRIM25 overexpression to induce NFkB signalling and the restriction we are measuring (Figures S1F and 3K). Importantly, TRIM25 is still antiviral in TBK1 KO cells (Figures 3I and 3J), which we interpret as an indication that the activation of classical signalling pathways is not involved in this phenotype.
- Regarding Figure 2, we would like to redirect the reviewer to our response to point 8.
- We disagree with the reviewer regarding the levels of IFNs used in the experiments presented in this manuscript. As now mentioned in the introduction, as response to this reviewer’s specific comment #3, despite the role of VP35 and VP24 on immune evasion, upregulation of both type-I and type-II IFNs is significantly observed in moderately ill and survivors of Ebola virus infection (Hutchinson, 2007 and McElroy 2016). The levels reported ranged from 2-3 log pg/ml of IFN-b for McElroy et al., and 50-10,000 pg/ml of IFN-a for Hutchinson et al. These quantities are well in range of the quantities used in our experiments which are below 3 log pg/ml. Thus, as we mentioned in the manuscript, we envisage TRIM25 as a major component of the innate immune response to viral infection. Given reports as the ones referred above, we believe this to occur early in the infection of an individual, with a simultaneously deployment of viral antagonistic factors. The outcome of such events would necessarily be complex and context dependent.

2) There is inaccurate description of EBOV VP35 as co-factor for NP. On at least two occasions (line 76 and 306) the authors state VP35 is NP’s co-factor. VP35 is the polymerase (L) co-factor (Tchesnokov et al. 2018, Muhlberger et al. 1999). VP35 does act as a chaperone for NP (Leung et al. 2015, Kirchdoerfer et al. 2015) and links NP and L to facilitate transcription and replication of the EBOV genome. Also, when the authors describe viral ribonucleoprotein they describe it as being composed of NP, VP30, VP35, and L, but this is the composition of the viral transcription complex. The viral RNP is composed of, minimally, NP, VP35, and VP24 (Huang et al. 2002).

We thank the reviewer for getting our attention to the misrepresentations of the literature in our manuscript. We have now updated the referred sections as suggested (lines 83-89).

Regarding the proteins NP, VP30, VP35 and L, this group of proteins is often referred interchangeably as components of the nucleocapsid or of the ribonucleoprotein complex as referred to in e.g. Sugita et al., 2018, Nature; Hoenen & Feldmann, Nat Rev Microb, 2019. Independently of that, we recognise that we didn’t properly refer the presence of VP24 in the complex, which we have now corrected (Line 87-88).

3) In regard to the authors’ over-expression experiments, for several immunoprecipitations, the protein being pulled down is clearly not equal which may bias their interpretation of the results. For example, in Figure 5A, the authors are pulling down for endogenous TRIM25 and the levels of the input of TRIM25 are higher in the lanes expressing nucleoprotein than GFP or VP35 alone. Although the levels of TRIM25 in the PD are not that different, the levels of VP35 in the input are also lower than in the other sample. This is important since the authors are claiming that TRIM25 does not bind to VP35 in the absence of NP. The Figure S3A also has a similar issue where TRIM25 is not expressed as highly in the VP35 only sample as the NP only and NP + VP35 lanes (Input). Although the IP in the case of Figure S3A was for NP rather than TRIM25, it is odd that the authors would have obvious differences in expression in the corresponding samples in two different experiments. Similar issues of differences in expression of controls are also observed in Figure S3D, S3E, and S3F. The authors should do an immunoprecipitation for VP35 for NP, TRIM25, and NP + TRIM25 to be certain that there is interaction between VP35 and TRIM25

We agree with the reviewer regarding the different levels of some proteins visible in the immunoprecipitations. This was something we have observed from the very start of the project, in which the co-expression of TRIM25 and NP frequently led to apparent increase in levels of these proteins, likely due to some stabilisation and/or conformational change as consequence of the interaction. However, a well-known biological attribute of antiviral TRIM family proteins (see literature for both TRIM5 and TRIM21 for example) is that they tend to have short half-lives until they meet their targets. Upon such interaction, the TRIM is stabilized and undergoes autoubiquitination. So, in fact the increased protein levels of TRIM25 is indicative of a functional interaction.

On the other hand, we followed the reviewer’s suggestion to perform the immunoprecipitation with a VP35 antibody, using its known interaction with NP as control (as seen in the right panel of figure 5A). We changed the composition of the IP buffer according to what was published elsewhere for this interaction, and not only were able to see the VP35-NP interaction, as expected, but were also able to precipitate the TRIM25 protein independently of the presence of NP, although it was stronger when NP was transfected. This observation reinforces the evidence that TRIM25 can interact with the vRNP, while not having an impact on the main finding of this figure, that is the clear interaction of TRIM25 with NP.

4) The authors also do not show evidence that TRIM25 directly ubiquitinates NP. They rely on over-expression of TRIM25 or HA-Ub, and they do not interrogate which lysine residues or region of NP is ubiquitinated or perform an in vitro ubiquitination assay to clearly demonstrate TRIM25 is directly ubiquitinating NP. No endogenous ubiquitination is shown either.

We start by answering to the last point of this comment to clarify the experiment shown in figure 5F. In this experimental set up we are in fact showing ubiquitination of NP with endogenous levels of ubiquitin. The observable smear above the NP band in cells transfected only with TRIM25 disappears upon treatment with USP2, an enzyme that cleaves most ubiquitin linkages. This is the clearest evidence that NP is ubiquitinated in the presence of TRIM25 by endogenous ubiquitin and the use of deubiquitinases to prove it is viewed as a gold standard by many in the field. We have found this assay far more robust than commercial Ub-specific antibodies because of sensitivity issues.

As just mentioned, we showed clear evidence that NP gets ubiquitinated in the presence of TRIM25. The molecular mechanisms associated with this phenotype are of major interest for us and we have planned to follow-up this route of investigation, but such evolved biochemical studies are, we feel, beyond the scope of this submission.

With respect to mapping the lysine residues in NP, there are 38 lysines in the protein. We have preliminary data that there are mixed Ub linkages (K27, K33, and K63 predominantly) that we intend for a follow up paper. This suggests that there will be multiple lysine acceptor residues, and this will take extensive further study to investigate. While interesting to us, this is beyond the scope of the current study and would not materially change its conclusions.

**Specific Comments:**

1) Lines 65-67: The paper referenced regarding evidence for bat hosts of EBOV is a serology-based studies. Stronger papers to support this statement in regard to EBOV include the finding of Bombali virus in insectivorous bats (ex. Goldstein et al. 2018).

We have taken the suggestion of the reviewer and swapped the reference of the filovirus serology-based study with the suggested manuscript (Goldstein et al. 2018) and other showing the genetic identification of a filovirus in dead bats in Europe (Negredo et al, 2011).

2) Lines 76-89: In describing the vRNPs of EBOV the authors make a couple errors, as described above, in their reporting of VP35’s roles and including VP30 as an essential vRNP component while leaving out VP24.

As referred above, we have included the suggested correction in the mentioned sections of the manuscript (lines 83-89).

3) The authors do not discuss the host’s IFN-I response to EBOV or filovirus infection in the introduction. Considering the phenotypes they observe in the context of the trVLP system are highly reliant of IFN-I pre-stimulation, it is important to provide this information.

We thank the reviewer for the suggestion. We have updated the introduction accordingly (Lines 107-114).

4) It would be beneficial to include the Figure S1A in the main text and/or give a brief explanation of the trVLP system (specifically the minigenome component itself) in-text. The Figure S1A itself is also difficult to read.

We followed the suggestion of this reviewer and updated this panel in a way that we hope it’s now clearer and moved it to the main Figure 1. We also added to the text a brief description of the EBOV trVLP system as suggested (Lines 136-148).

5) Figure 1B: It is unclear why this data was presented on a log scale when the output is reported as a percentage. There is probably a more substantial effect of IFN-I on EBOV particle assembly that would be easier to see with a linear scale, especially considering the statistical test indicates a significant difference.

We have changed the scale representation in this figure as suggested by the reviewer.

6) In the over-expression experiments for reporter assays (Figure 2A) and throughout the paper, the authors do not include western blots to monitor over-expression of TRIM25.

The expression levels of proteins in the context of the signalling assays were not initially included as they were regularly shown in the context of the CRISPR cells characterization and/or pull-down experiments. We believe that this would make it redundant particularly given the considerable size of many of the figures, and because they already included several controls (e.g., CRISPR cells or mutants) to consolidate the specificity of the phenotypes. However, to accommodate the suggestions of the reviewers, we have now included expression data for most of the mentioned experiments.

7) Lines 190-193 and Figure 2A: It is unclear why there would be an equivalent difference between p1 and p2. The authors should provide some explanation. How are the authors able to control how many trVLPs are added from the p1 supernatants to p2? If there is no way to control this, then I would expect there to be a larger difference in p2 than p1.

In the methodology used in this manuscript, we transferred a given volume (equal to all experimental wells withing a given experiment) from p1 supernatants into fresh p2 HEK293-TIM1 cells pre-transfected with the vRNP components. Viral inputs into P1 were always standardised as the volume of trVLP prep that gives 1x10^6^ RLU in the absence of ISG overexpression which we have determined to be in the linear range. Regarding the point raised by the reviewer, we would like to clarify that there is no transfected TRIM25 in p2 cells, thus we would not exactly necessarily expect a cumulative effect between p1 and p2. Given that the fold difference in viral RNA levels (on TRIM25 v GFP transfected cells) is very similar in the supernatant when compared with the intracellular levels (Figure 2C), we were not surprised that the phenotypes in p2 are equivalent to the ones in p1.

8) Figure 2I: As stated above, it is unclear why you observe a difference in trVLP production in p0 TRIM25 knockout cells when you do not see a difference between wt and TRIM25 knockout cells in the absence of IFN-I treatment in Figures 2E, F and 2G.

We understand the reviewer’s point, but these experiments are not directly comparable. In Figure 2I we have an experimental set up in which the trVLPs are exclusively being generated (in p0) and there are no incoming virions, as it occurs in the cells infected with trVLPs in the absence of IFN-I. In this p0 step, trVLPs are formed upon transfection of the viral RNA minigenome, the T7 polymerase that will guide the initial transcription and plasmids expressing the viral proteins not codified via the genome - NP, VP35, VP30 and L.

The p0 results on this panel can potentially be explained with the hindsight of the later findings. For example, TRIM25 might be able to interact with freshly synthesised NP and VP35 proteins, where endogenous levels can be enough to have deleterious effects on the particle formation which are absent in the CRISPR cell line. On the other hand, TRIM25 is a well-known cofactor of ZAP which targets viral RNAs (as we have also shown). In the same line of the special circumstances that is the artificial formation of trVLPs in p0, endogenous ZAP might have some level of access and restriction to freshly synthesised viral RNAs which would likely not occur in the absence of TRIM25. Finally, the p0 cells are lysed 48 hours after transfection, while the p1 cells are harvested 24 hours post-infection, opening the further possibility that additive effects on the minigenome would be more visible on p0 rather than in p1.

9) Lines 211-214: The authors should include a reference for this statement

As suggested, we added two references to this statement (Cadena et al. 2019; Oshiumi et al. 2013).

10) Figure 4: Across the panels, it is not consistent whether ZAP-L has an effect in the absence of IFN-I. It’s unclear if this is due to the method of normalization or the lack of an effect in the absence of IFN-I. If it is due to the method of normalization, the authors explain more clearly.

The RNA is normalized to the RNA in the LacZ-CRISPR control cells where we saw no detectable difference in levels with the ZAP KO cells, whereas the read out of trVLP replication/gene experession (RLuc) shows a small 2x increase at baseline. This is not necessarily unexpected. ZAP can mediate both degradation and translational repression of viral RNAs and what controls this is not at present known. The RLuc readout will be a composite of these two non-mutually exclusive mechanisms but the RNA readout only measures viral abundance and not function.

11) It may be useful to include supplementary figures S4I and S4J in the main text.

We appreciate the reviewer’s suggestion and have considered this option. However, given the large number of figures that the manuscript has already, and the space limitations in the associated Figure 7, we have at the end opted to keep these panels as supplementary.

12) Figures 5 and 6 were flipped in the document.

Thank you for getting our attention to this. We have now corrected the order of these figures in the pdf generated on submission.

13) TRIM25 can bind both dsRNA and ssRNA (Sanchez et al. 2018), but RNAse A will only work for single-stranded RNA. Since there are dsRNA replication intermediates of the EBOV minigenome the experiment should be repeated with RNAse III which can cleave dsRNA.

We thank the reviewer for highlighting this for us. We followed the suggestion and have performed another pull-down experiment in the presence of RNAse III and showed that the NP interaction with TRIM25 is also not sensitive. This experiment is shown in the right panel of Figure S3B.

14) Several western blots have background for NP (ex. Figure 5E) in the GFP pull-downs.

Regarding the specific case of Figure 5E, we would like to highlight the fact that this experiment was performed in HEK293T-TIM1 cells, where endogenous levels of TRIM25 were present. Thus, in the presence of Ub-HA, the signal observed in the HA-Ub pull-down fraction for NP, in GFP transfected cells, is likely due to direct action of TRIM25 endogenously existent in the cells, or via another cellular factor.

15) Figures 5E and S3D: The way the experiment is laid out, it is difficult to appreciate a difference in the ubiquitination of NP with the addition of TRIM25. It would be more effective to have the HA-Ub IP with TRIM25 WT + NP and TRIM25 catalytic mutant/RING domain deletion in adjacent lanes. In the supplementary figure there is less HA-Ub expressed and pulled down in the samples with the TRIM25 mutant as well further complicating an accurate interpretation.

The first point raised by the reviewer is not clear to us. We might be misunderstanding the comment, but the difference in ubiquitination of NP with the addition of TRIM25 WT seems clear to us, either in the Pull-down performed with NP in figure 5E, as with the pull-downs performed with anti-HA-Ub in both Figure 5E and S3D. Thus, we don’t understand the suggestion to change the layout of the experiment. However, we would be happy to be clarified and address this if required.

As explained in point 14, these set of experiments were performed on HEK293-TIM1 cells where the endogenous levels are arguably enough to promote a residual level of NP ubiquitination when co-transfected simply with GFP and HA-Ub. The over-expression of ∆RING has been shown to act as a dominant-negative (Zheng et al, 2017) what might explain the absence of the so-called “NP background” in the respective pull-down fraction. This can also explain the reduced level of total HA-ubiquitin pulled-down upon ∆RING transfection when compared with the GFP fraction. Furthermore, TRIM25 is a E3 ligase and overexpression will increase the amount of cellular proteins being ubiquitinated and therefore Ub-HA would appear more apparent in the input. Although the slight lower levels of total Ub-HA observed in the experiment, given the nature of TRIM25 WT and ∆RING, we believe that the data clearly shows a TRIM25 WT-mediated ubiquitination of NP which can’t occur when the RING domain of this protein is absent.

16) Looking at endogenous levels of NP ubiquitination in wild-type versus TRIM25 knockout cells following infection with the trVLPs would be a clearer experiment. Especially if the knockout is rescued with wild-type or catalytic mutant TRIM25.

We agree with the reviewer that this experimental setup would be the ideal to further consolidate the ubiquitination data shown throughout the manuscript. Unfortunately, this is not possible to achieve due to technical limitations. The amount of NP exclusively present in incoming trVLPs is difficult to detect by Western blot and/or to pull-down with the antibodies we have available, which together with the poor performance of the tested anti-ubiquitin antibodies in our experimental conditions, makes this experiment a step too far for the potentialities of the EBOV trVLP system.

However, we would like to reinforce the notion that, although we observe a correlation between NP ubiquitination and antiviral activity, we don’t argue to have fundamentally proven that this is essential from TRIM25-mediated restriction of EBOV. In fact, in the discussion, we hypothesised that the multimerization of TRIM25 on EBOV helical vRNP could be driver to its disassembly and thus recruiting other RNA-directed antiviral factors and sensors (lines 538-553). This is also the case for other antiviral TRIMs where the autoubiquitination of the TRIM is essential, but the that of the target protein (i.e. HIV-1 CA in the case of TRIM5) is secondary.

17) Figure 6A: It is unclear what groups are being compared for statistics.

We consider it important to compare whether a given TRIM25 mutant has a significant antiviral activity compared to the expression of the negative control (stars on the bars), as well as whether the antiviral potency is significantly reduced compared to the wild type TRIM25 (stars above the bars). This is clarified in the figure legend.

18) Lines 342-345: The authors need to include citations regarding the TRIM25 RNA binding sites

We have added Choudhury et al, 2017 and Sanchez et al, 2018 as citations to this sentence.

19) Figure 6B: it appears that the authors assume that the NP smear corresponds to ubiquitinated NP (NP-Ubn). This needs to be shown with anti-ubiquitin antibody against endogenous Ub.

The experiment in Figure 5F was performed with endogenous levels of Ubiquitin, and it shows the collapse of the smear from immunoprecipitated NP by the ubiquitin specific protease USP2 demonstrating that this is direct ubiquitination of NP. We have found this assay far more robust than commercial anti-Ub antibodies.

20) Figure 6C: The pull down for TRIM25 is not equal. Since the authors are claiming that the SPRY deletion mutant loses interaction, there needs to be an equivalent amount of TRIM25 expressed.

We don’t necessarily agree with the analysis of the pull-down data by the reviewer. We don’t think the level of ∆SPRY mutant pulled down is in any way different from the one obtained with TRIM25 WT. Therefore, we still stand by our interpretation that the deletion of the SPRY domain, as well as the 7KA mutation, reduce the interaction of TRIM25 with EBOV NP.

21) Lines 369-371: It would be helpful if the authors framed the 5% of cells with NP-TRIM25 interactions in the context of the percentage of cells that are NP positive.

This point was also raised by reviewer #2 and we acknowledge that the text didn’t clearly transmit the information as we intended. We have observed that in the experimental conditions used in these assays – staining 4-6 hours post-infection with concentrated trVLPs and no transfected NP present - in HEK293T LacZ CRISPR-TIM1 cells we could detect NP-positive foci in around 5% cells, from which almost the totality of these cells presented a re-localisation of endogenous TRIM25 to NP-enriched foci. We have altered the text to make this description clearer (lines 397-399).

22) Lines 439-445: Based on the data presented in the paper, I would argue that TRIM25 is recruited to viral RNA rather than NP since the RNA binding mutants also have decreased interaction with NP. The authors do state that RNA binding regions of TRIM25 could be influencing the conformation of TRIM25 SPRY and thus binding to NP, but further experiments are needed to clarify this aspect of their proposed model.

We agree with the reviewer to a certain extent in that the RBD is essential for antiviral activity. However, the RBD-mutant can still interact with NP in pulldowns. As we say in the discussion, we propose that TRIM25 sees the viral RNA in the context of the helical NP array. The RBD is essential for activating the Ub-ligase activity of TRIM25, which we hypothesise is the driving force of disrupting the NP/RNA interaction and revealing the genomic RNA to ZAP. Testing this proposal will be a priority for follow up studies.

23) Lines 449-451: It is unclear why the authors mention that “until recently” TRIM25 is positive regulator of RIG-I yet later in their discussion point to TRIM25 being targeted by viral proteins (lines 560-562) in regard to their blocking TRIM25 from activating RIG-I.

The study of the action of K63-Ubn on RIG-I has been thorough and there is a detailed understanding of it. However, the debate remains arguably open regarding the how and when Ub is added to RIG-I and which precise E3 ligase involved. Several previous studies reported that TRIM25 and RIPLET are two essential E3 ligases important to RIG-I signalling (e.g. Gack et al 2007, Oshiumi et al 2010), while a more recent study suggested that RIPLET and TRIM25 sequentially act on RIG-I upon viral RNA engagement (Oshiumi et al, 2013). Finally, it was proposed by Cadena et al (2019) that RIPLET is sufficient to ubiquitinate and activate RIG-I, what is behind the use of “until recently” in our sentence.

As the reviewer will understand, it’s not the goal of this manuscript to enter in this debate, particularly because the latter manuscript (Cadena et al, 2019) doesn’t state that TRIM25 is not able to ubiquitinate the signalling domain of RIG-I. Furthermore, we don’t believe that this most recent finding puts in doubt all the work performed by others showing that viral proteins specific target TRIM25 as a mechanism to evade RIG-I mediated detection. However, we accept that some of these findings might have another layer of interpretation in the light of the recently described function and importance of RIPLET.

We have updated the above-mentioned sections to better represent the present knowledge about the topic (lines 477-483).

24) Lines 549-552: The contribution of MAVS to TRIM25’s proposed antiviral function is not well explained.

We followed the advice of the reviewer and tried to make this section clearer in the discussion of the manuscript (lines 586-601).

25) Line 569: The authors should include a citation

As suggested, we added citations regarding bats being the putative reservoirs of EBOV and other filoviruses (Goldstein et al, 2018 and Leroy et al, 2005)

26) Lines 856-858: The authors use digital droplet PCR and do not need a standard curve to determine DNA copies, however the authors need to include validation for strand specificity or provide reference to a paper that have previously done the validation with these primers.

The strand-specific reverse transcription was performed according to the approach presented by Kruse et al., 2018. Accordingly, for the cDNA synthesis from EBOV trVLP -vRNA we have used a reverse primer targeting the trailer region of the genome (EBOV -vRNA RT), while to produce cDNA from the viral +cRNA we used a forward primer (EBOV +cRNA RT) targeting the same region. We have added the reference and a more complete description of the procedure to the methods section.

**Reviewer #2:** Missing changing titles and y-axis in the figures

1. The majority of the data presented in this paper is the result of a few kinds of experiments repeated multiple times with varying conditions. For example, trVLP replication/transcription in p1 and p2 cells is assessed via luciferase reporter assay under conditions that include IFN treatment, ISG over-expression, gene knockout, etc. Similarly, trVLP replication/transcription is also assessed via RT-qPCR under similarly varying conditions. This means that much of the data in this paper is (or should be) consistent from experiment to experiment, outside of the effects of whatever variable is changed. However, it is confusing that none of the data is presented consistently. For the sake of clarity, it would be extremely helpful if the authors could revise their figures to standardize axes, titles, and assays throughout their paper. For example:

a. trVLP assays are depicted in Figures 1A, 2A, 2E, 2G, 2I, 3C-D-E, 3G, 3I, 3J, 4A, 4C, 4E, 4G, 4I, 5D, and 6A; however, none of these figures use the same y-axis range. Indeed, in the case of 4A, the y-axis range differs between the two panels in the same sub-figure. As a result, the magnitude of the differences between conditions within an experiment/figure cannot be easily compared with the results of a different experiment/figure. In some cases, such as in Figures 3C and 3G, the narrow y-axis range effectively increases the appearance of a difference in magnitude when the actual differences is quite small. Since all of these data are based on the same fundamental experiment, it seems appropriate to use identical y-axis ranges for all.

b. The figures depicting RT-qPCR data are also confusing at times. The methodology of Figures 1 C and D are ostensibly identical to that of Figure G, yet the axis labels and order of the data panels differs. Is it necessary to apply a different label to the vRNA supernatant data in Figures 1D and 1G? Is it necessary that these labels differ from the ones used in Figure 1C and the other two panels in Figure 1G? The presentation of data is also inconsistent in Figure 2F, 4D, and 4H. Please clarify.

c. Similarly, the y-axis ranges in Figures 7B-F and H should all be the same.

We thank the reviewer for the thorough revision of our figures. We agree with it and have now updated the figures accordingly to the suggestions presented in a-c. However, please notice that the y-axis values are different for experiments performed in HEK293T- compared with U87-MG-based cell lines due to the dynamic range of the RLU values that were possible to obtain in the latter (please see comment to point #3 of Reviewer #2). However, even so, we felt that it was more representative to present the RLU values rather than a percentage of control.

2. In Figure 2C, strand-specific RT-qPCR used to quantify the differences among vRNA, cRNA, and mRNA, but this was not done for any of the other RT-qPCR assays. Why?

We used the strand-specific RT-qPCR to specifically address the impact of over-expressing TRIM25 or ZAP in the levels of vRNA, cRNA and mRNA. This can be observed not only in Figure 2C, as the reviewer correctly highlighted, but also in figures S2A (for ZAP) and S4D (for both TRIM25 and ZAP at different points).

In all these figures we observed that over-expression of TRIM25 or ZAP led to a similar level of decrease of all viral RNAs, without any of them being particularly targeted and/or affected. Given this lack of specific targeting, we considered that there was no need to do this analysis in all the other RT-qPCR assays and opted for the quicker and more feasible approach that allowed us the use of random primers to perform the cDNA synthesis, instead of strand-specific primers.

3. Can the authors specify the dose of trVLPs used in each assay? On Line 155 they state that “a fixed dose” of trVLPs was used, but the dose is not stated. On Lines 675, they state that between 5-50 ul of supernatants were transferred to p1 cells. Why would the volume change from experiment to experiment? Do differences in the inoculum volume affect the differences observed in each of the assays? Please clarify.

The term “fixed dose” on line 155 refers to the fact that the same quantity of EBOV trVLPs was used in all the plates used in the screen. For all experiments performed in HEK293Ts, including the screen, we used an inoculum that would give us a reading of 10^6^ RLU in untreated/control p1 cells. Given the nature of U87MG cells and their difficulty to transfect, we would only be able to reach these RLU levels by infecting the cells with high volumes of EBOV trVLPs which we didn’t consider as the best practice. Thus, the RLU levels in untreated p1 U87-MG cells are normally closer to 10^5^ RLU. During the long course of this work, we had to produce several preparations of EBOV trVLPs, which had naturally different viral titres. Our statement regarding the use of 5-50µl of EBOV trVLPs regards both these differences - batches and cell lines. We recognise that this statement was not clear and might be confusing. We have now changed the text and clarified the quantities of EBOV trVLPs used in the experiments as mentioned above.

Lastly, for the sake of clarification on the last point raised by the reviewer, we observed no differences in our phenotypes that were dependent on the inoculum volume.

4. One of the biggest weaknesses of this study is that the authors provide no evidence of TRIM25/ZAP-mediated restriction of authentic EBOV infection. Can the authors attempt to validate their results using authentic virus? Such an experiment would greatly enhance the relevance of the conclusions, in addition to demonstrating the “translatability” of the trVLP modelling system. It is understandable if this kind of experiment is not possible, since it would require access to a high-containment laboratory. However, the authors should at least acknowledge this shortcoming.

The authors recognise that the lack of experiments performed with full-length EBOV is a limitation of this study. As the reviewer recognises, the unavailability of an accessible high-containment laboratory in the vicinity of our main site was a major block to perform these experiments. Also, COVID-related restrictions and availability of personnel at the site of collaborators did not allow us to perform these experiments in a safe and timely manner. However, one should not forget that Muller S et al. (2007) showed that over-expression of ZAP restricts the replication of full-length EBOV. Although our data suggests an alternative mechanism, this paper is supportive evidence that our phenotypes observed with EBOV trVLPs might be translatable to the full-length virus.

We added a sentence at the end of our discussion mentioning the absence of these experiments as a shortcoming of our manuscript (lines 498-502).

**Reviewer #3**:

1. Figures 2 and 3: Some experiments were performed with HEK293T or U87-MG cells alone, although the authors have established the CRISPR/Cas9-based KO clones for key factors in both cell types.

Through the manuscript we took the option that experiments that included pre-treatment with IFNs should be done in U87-MG cells, as they were the ones more sensitive and responsive to this treatment. However, these cells are not the easiest to manipulate/transfect and therefore we chose to use the more robust HEK293T cells to experiments that only included transfection.

As explained elsewhere with more detail (e.g., the reply to minor issue #2 of reviewer #3), we were aware that transfection of some ISGs might itself trigger different signalling pathways that lead to the expression of various interferons. This is particularly relevant in the context of U87-MG, as we wanted to minimize the contribution of the consequential expression of other ISGs, while focusing on the specific antiviral effect of the candidates we were studying.

Thus, by doing the experiments in this setup, we believe to have addressed the role of different proteins in the context of their over-expression trying to answer the questions “Do high amounts of a given protein, in WT cells or KO cells, have an impact on the virus?”. While in the context of pre-treatment with IFNs we tried to address if “Endogenous levels of a given protein have any role in the IFN-mediated restriction of EBOV trVLP”

2. Figures 2, 3, 4, and 7: I suggest that the authors add the data showing the exogenously expressed genes.

This aspect was also raised by reviewer #1 on specific point 6, and we have now addressed it to the best of our possibilities (please see comment above). As mentioned, we felt that the representation of these protein levels would be redundant considering the over-expression data shown elsewhere (e.g. pull-downs), the internal controls used, and the present size of the figures. Also, the impact of the luciferase assay lysis buffer on the quality of the blots led us to the initial decision of not including such analysis. However, we have now included expression data for most of the mentioned experiments as appropriate.

3. Figures 5B and 7A: How do the authors set up the experiment condition? The information about the incubation time in the figure legend and the text is not consistent. The authors must provide the precise and detailed methodology in the manuscript. According to the pattern of NP signals in the images, they seem to be inclusion bodies instead of incoming single vRNPs.

We apologise for the oversight and hope to have made the experimental conditions clearer throughout the text – this includes the main text, methods and figure legends.

For clarification, cells on Figure 5B were stained 24 hours after transfection. This experiment was designed to address if there was any relocation of endogenous TRIM25 upon over-expression of NP, with or without the presence of other proteins that compose the viral RNP.

For Figure 7A, cells were infected with concentrated EBOV trVLPs and stained 4-6 hours post-infection. One should highlight that no transfection of NP, or other RNP components, was performed in this experimental setup. Thus, there is no possibility that new transcription of NP protein could occur (NP isn’t encoded by the trVLP genome), and all the detection of NP in this experiment must be coming from incoming vRNPs. We can’t formally rule out that we are imaging inclusion bodies but, even if that is the case, we were still able to observe endogenous TRIM25 co-localising with EBOV NP that is only possible to have its origin in the incoming vRNP. Furthermore, the fact that we can’t see this re-localisation of endogenous TRIM25 in NPC1 CRISPR cells, in which no infection can occur, offer the setup the required control and specificity.

**Part III – Minor Issues: Editorial and Data Presentation Modifications**
Please use this section for editorial suggestions as well as relatively minor modifications of existing data that would enhance clarity.

**Reviewer #1**: (No Response)

**Reviewer #2**:

1. Can the authors indicate (in the figure legends or Materials and Methods, for example) the amounts of IFN that were used in each assay instead of referring to “increasing amounts of IFN”?

We have indicated in the material & methods the precise amounts of the different IFNs used in the pre-treatments before infection with EBOV trVLPs. We have done the same for the T705 compound (lines 726-729).

2. Are the differences in RNA levels in Figure 4D significant?

Yes. The differences in RNA levels are significant in the three panels of figure 4D. We have now added the statistical analysis to the figure.

3. On Line 370, the authors state that 5% of transfected cells exhibited NP foci that colocalized with TRIM25. Does this mean that 95% of the cells expressing NP did not exhibit the same colocalization? What might this say about the ability of TRIM25/ZAP to act as an EBOV restriction factor?

No, in fact we meant that only 5% of the cells were infected with the EBOV trVLPs as indicated by the detection of the viral NP protein – please note that there was no transfection of NP in this experimental setup. TRIM25 colocalised with NP in all infected cells. We have now changed the text to make this point clearer (lines 397-399).

**Reviewer #3**: 1. Please describe the technical terms in the text and the figures in a consistent way (e.g. TIM-1 or TIM1, TRIM25 or Trim25, EBOV or EboV, hours post-infection or hpi, HA-Ub or Ub-HA).

Figures and text have been updated accordingly.

2. Please indicate the origin of cell lines in the materials and methods. Please explain the reasons that the authors used HEK293T cells for ISG screen although U87-MG cells exhibited more significant sensitivity to IFN-I (Figure 1C and D).

Catalogue references from the parental cell lines used in this study were added to the material and methods section.

As stated in the manuscript, we agree that U87-MG cells are more sensitive to pre-treatment with IFN-I, and that EBOV is particularly sensitive to IFN-I in these cells. Although we also clearly observed IFN-I-mediated phenotypes in HEK293T cells, the dynamic ranges observed in the experimental readouts have made U87-MG the cell line of choice for setups that include treatments with IFN-I. However, these cells are quite difficult to manipulate and are not easily transfectable. Therefore, for the ISG over-expression screen, and other assays that didn’t involve IFN-I treatments, we opted for HEK293T cells as they perfectly support the EBOV trVLP system, while being easier to transfect and generally more robust.

Furthermore, it is known that over-expression of certain ISGs can activate signalling pathways that lead to the expression of IFN-I. This could be considered as a confounder in the context that our objective was to identify ISGs that were directly antiviral against EBOV. Thus, using a cell line highly responsive to IFN-I would increase the probability of identifying ISG’s being antiviral not because they would directly act on EBOV, but because their over-expression was promoting the production of other antiviral effectors. Although making this screen on HEK293Ts doesn’t exclude these possible occurrences, it makes it less likely when compared with U87-MG cells.

3. Line 237, what is RLR signalling?

RIG-I-like receptor signalling. Now explicitly written in the text. (Line 257)

4. Line 298, Figure S4I and S4J must be S2I and S2J, respectively.

Thank you for spotting this. We have now corrected it accordingly.

5. Figures 4G, 4I, S2D, and S2J: Please add the results of statistical analysis.

Statistical analysis added accordingly.

6. Figure1E-G: Please highlight TRIM25, IRF1 and BST2 in the figure.

We have highlighted these ISGs as suggested.

7. Figure S1A, lower panel: The budding virions seem to be the vRNPs.

We have changed the “design” of the virions in this figure (now Figure 1A) so they are distinguishable from the vRNPs represented elsewhere.

8. Figure 7C and H: Please modify the color of legends for NP-associated RNAs.

We have modified the legends within these figure panels as suggested.
