# Supplementary material for: TRIM25 and ZAP target the Ebola virus ribonucleoprotein complex to mediate interferon-induced restriction
Source: PLoS Pathog. 2022 May 9;18(5):e1010530. doi: 10.1371/journal.ppat.1010530 (PMC9119685; doi:10.1371/journal.ppat.1010530)
Supplement: Attachment — Submitted filename: Galao RP_Response to Reviewers_April 2022.docx [file ppat.1010530.s010.docx]

**Responses Reviewer's Comments**

**Part I - Summary**
Please use this section to discuss strengths/weaknesses of study, novelty/significance, general execution and scholarship.

**Reviewer #1:**

Overall, the Galao et al. resubmission of “TRIM25 and ZAP target the Ebola virus ribonucleoprotein complex to mediate interferon-induced restriction” is much improved from the initial submission, and the majority of comments were addressed sufficiently. However, two concerns remain to be addressed as described below. The main concern that must be address is definitive evidence that NP is ubiquitinated. One of the main conclusions is ubiquitination of NP and this has to be demonstrated by probing for endogenous ubiquitin. This is consistently done in the ubiquitin field, and there is no reasonable explanation on why technically it is not possible to use antibodies against endogenous Ub. Furthermore, TRIM25 is also responsible for ISGylation, and has also been reported to produce unanchored ubiquitin chains. The methods that the authors use cannot distinguish between these, thus they need to include an experiment using endogenous ubiquitin and an ubiquitin-specific antibody.

**Reviewer #2:**

In the revised version of their manuscript, Galao and colleagues have adequately addressed almost all of the reviewer comments. The result is a much improved paper, particularly with respect to the presentation and description of the data. I have only a few minor comments that might further enhance clarity.

**Reviewer #3:**

The authors have incorporated some comments from the first round of review; however the following key issues remains. Please refer to my specific comments in Part II – Major Issues.

We thank all the reviewers for their thorough revisions and contributions to the improvement of the manuscript. Below we present a point-by-point response to the latest comments, which we hope to sufficiently address the reviewer’s concerns and suggestions.

**Part II – Major Issues: Key Experiments Required for Acceptance**

Please use this section to detail the key new experiments or modifications of existing experiments that should be absolutely required to validate study conclusions.

Generally, there should be no more than 3 such required experiments or major modifications for a "Major Revision" recommendation. If more than 3 experiments are necessary to validate the study conclusions, then you are encouraged to recommend "Reject".

**Reviewer #1**:

*Overall, the Galao et al. resubmission of “TRIM25 and ZAP target the Ebola virus ribonucleoprotein complex to mediate interferon-induced restriction” is much improved from the initial submission, and the majority of comments were addressed sufficiently. However, two concerns remain to be addressed as described below. The main concern that must be address is definitive evidence that NP is ubiquitinated. One of the main conclusions is ubiquitination of NP and this has to be demonstrated by probing for endogenous ubiquitin. This is consistently done in the ubiquitin field, and there is no reasonable explanation on why technically it is not possible to use antibodies against endogenous Ub. Furthermore, TRIM25 is also responsible for ISGylation, and has also been reported to produce unanchored ubiquitin chains. The methods that the authors use cannot distinguish between these, thus they need to include an experiment using endogenous ubiquitin and an ubiquitin-specific antibody.*

Since TRIM25 is both an ubiquitin and ISG15 ligating enzyme (3, 5, 6), it is essential that the authors provide definitive and direct evidence that NP is ubiquitinated versus ISG15ylated. Further, USP2 is a cross-reactive enzyme that is able to cleave both ubiquitin and ISG15 from modified proteins (7), thus the use of this particular deubiquitinase cannot be used to confirm that the modification to NP is ubiquitin. The authors must perform pull-down assays for NP (in denaturing conditions), and immunoblot for endogenous ubiquitin. Show a blot with ubiquitin-specific antibody, specifically in the absence of Ub over-expression. The author’s response to reviewer #1’s specific comments 16 and 19 are thus insufficient responses. Commercially available antibodies against endogenous ubiquitin are used extensively and are of excellent quality. There is really no reasonable explanation on why not to use antibodies against endogenous ubiquitin. This is standard in the field.
If the issue is NP, Commercial NP antibodies are available that may work better than the antibodies generated in-house (<https://www.kerafast.com/item/1007/anti-zaire-ebola-virus-nucleoprotein-1e6-antibody>). Figure 5E shows perfectly good pull-down for NP, so no reason why not to immunoblot for endogenous ubiquitin in the absence of over-expressed Ub. Please add molecular weight markers to all blots.

We thank the reviewer for the supportive comments regarding the alterations previously done on the initial manuscript and hope to have convincedly addressed the latest comments with the newly performed experiments and the answers below.

We agree with the reviewer and accept that the conclusions of the manuscript would be better supported by showing the ubiquitination of NP in the absence of exogenously expressed ubiquitin and probing for endogenous levels of the latter. For that, we performed the experiment suggested by the reviewer and show in the new Figure 5G (+lines 367-374) that following a pull-down for NP we can detect an increased signal for endogenous ubiquitin in the samples that were co-transfected with NP and TRIM25, in comparison with samples transfected only with one of these proteins. Of relevance, the increased endogenous ubiquitin signal corresponds to the expected molecular weight of NP.

We also immunoblotted the samples for ISG15 and were unable to detect any signal for ISGylated proteins (not shown but can be added to the figure if the reviewer requests us to do so). At least in these cells and experimental setup, TRIM25 doesn’t seem to mediate ISGylation of NP. However, we can’t exclude a TRIM25-mediated ISGylation of NP in conditions where ISG15 is present or induced. We now address this in the manuscript (discussed in lines 564-566).

**Reviewer #2:** None

**Reviewer #3**:

1. Although the authors advocated that they used U87-MG glioma cell line because of its sensitivity and responsiveness to the IFN signaling, glioma cells are not major target cells in the context of EBOV infection. In fact, quite few EBOV studies used this cell line in the literature. As the authors mentioned in the introduction (lines 111-112), the myeloid cell lines must be more appropriate to assess the roles of TRIM25 and ZAP in EBOV life cycle.

As we mentioned previously, and the reviewer here refers, we agree that myeloid cell lines would be more appropriate as a cellular model to our studies. However, the nature of the trVLP system with its strict requirement for the transfection of the EBOV RNP components, doesn’t favour its use on myeloid cell lines because of cytoplasmic DNA sensing of the plasmids that would activate interferon and confound any results.

Although we accept the argument that U87-MG glioma cells are not a major target for EBOV infection, we still think that these cells are very useful and instructive in defining the role of TRIM25 and ZAP. U87MG have been used in several studies (with HIV for example) because they have little or no endogenous ISG expression and are thus a clean system for examining IFN effects on viral replication. Secondly, they support high levels of trVLP production and replication. As seen below and in the main Figure 1, IFN is antiviral against EBOV trVLPs not only in U87MG cells but also HEK293 cells – however the magnitude of the effect is more pronounced (left panel below). This limited range in HEK293 made our IFN/EBOV studies more challenging as you can see in the right panel below, where however we consistently obtained a higher EBOV trVLP titres in HEK293 TRIM25 KO cells compared with WT cells, we could not obtain more than 2-3 fold variations in contrast to the data we present in the manuscript.

2. The authors argued that theoretically transcription of NP protein would not occur and all the detected NP signals in this experiment must be derived from incoming vRNPs. However, I still don’t understand the NP signal pattern in Figure 7. The size of each vRNP is supposed to be approximately 100 nm in diameter and 1-2 µm in length. Detected NP signals are quite large for its predicted size in these images. Why?

The reviewer is correct in his assessment that the NP signals observed by us in trVLP-infected cells are too big to represent single vRNPs. However, as pointed out by us before, these cells are not transfected with NP-encoding plasmids, and thus the only possible source of NP are incoming trVLPs. Therefore, we would suggest that the NP signals observed do not represent single vRNPs, but rather accumulations of several vRNPs, since we use highly concentrated trVLP stocks for these infection experiments - We now refer this possibility in the main text (lines 409-411). However, it remains unclear whether this accumulation is driven by forces that usually result in the formation of inclusion bodies, or whether this accumulation is the result of a cellular defence mechanism driven by TRIM25. Importantly, while this phenomenon is peculiar, it does not invalidate our observations or conclusions regarding the antiviral activity of TRIM25.

On a more technical aspect, we might not be able to optimally permeabilise the endosomal/membranous structures within the cells which might not allow for the perfect staining of individual/smaller virions structures.

**Part III – Minor Issues: Editorial and Data Presentation Modifications**

Please use this section for editorial suggestions as well as relatively minor modifications of existing data that would enhance clarity.

**Reviewer #1:**

1) Please add Molecular weight markers to ALL immunoblots.

Molecular weight markers were added to all immunoblots.

2) In response to the author’s response to reviewer #1’s main point 3, their explanation for why the experiments were not repeated to ensure controlled expression between groups for IPs is insufficient. It is known that TRIM25 undergoes autoubiquitination (1, 2) and autoisgylation (3), but in these studies and others TRIM25 expression (over-expression and endogenous) does not seems to be influenced regardless of infection, capacity for autoubiquitination, or over-expression of other proteins (2-4). Further, they provide no citations to support their claims and simply state ‘see literature’. The conclusions are unlikely to be changed substantially by the differences in expression, but the paper could be improved by having experiments with equal levels of expression.

We accept that a stabilisation and/or increased expression of TRIM25 while undergoing autoubiquitination and/or mediating target-ubiquitination might not be a defining feature of this E3-ubiquitin ligase. However, there is evidence of it occurring even if that fact is not highlighted as such. For example, one can observe an increased expression of TRIM25 in the presence of the 2CARD domain of RIG-I on Figure 2B of the original TRIM25/RIG-I manuscript by Gack M, Nature, 2007 (attached below).

In any case, we followed the reviewer suggestion and redone our initial experiment showing the interaction between TRIM25 and EBOV NP while controlling the levels of both proteins (new Figure 5A). As expected, we obtained the same result and conclusions as shown in the previous version of the manuscript. Taking this into account, we feel that the experiments in Figure S3 are sufficiently clear to support our conclusions and no substantial improvement would come from repeating all the experiments.

3) In response to reviewer #1’s specific point 14, their explanation does not address the original comment adequately. The authors will need to show a GFP pull down the in TRIM25 knockout cells to demonstrate no NP pulldown background to support their claim in their response to the reviewer that it is due to endogenous TRIM25.

As suggested by the reviewer, we now show in the new Figure S3G that the background levels of EBOV NP being pulled-down with HA-Ub, on GFP transfected cells, is no longer present when the experiment is performed in TRIM25 KO cells. Pull-down of NP with HA-Ub is restored in these cells upon transfection of a CRISPR-resistant form of TRIM25. Together, this offers further evidence on the role that TRIM25 plays in the ubiquitination of EBOV NP.

References
1. Gupta S, Ylä-Anttila P, Callegari S, Tsai MH, Delecluse HJ, Masucci MG. Herpesvirus deconjugases inhibit the IFN response by promoting TRIM25 autoubiquitination and functional inactivation of the RIG-I signalosome. PLoS pathogens. 2018;14(1).
2. Choudhury NR, Heikel G, Trubitsyna M, Kubik P, Nowak JS, Webb S, et al. RNA-binding activity of TRIM25 is mediated by its PRY/SPRY domain and is required for ubiquitination. BMC biology. 2017;15(1).
3. Zou W, Wang J, Zhang DE. Negative regulation of ISG15 E3 ligase EFP through its autoISGylation. Biochemical and biophysical research communications. 2007;354(1).
4. Gack MU, Albrecht RA, Urano T, Inn KS, Huang IC, Carnero E, et al. Influenza A virus NS1 targets the ubiquitin ligase TRIM25 to evade recognition by the host viral RNA sensor RIG-I. Cell host & microbe. 2009;5(5):439-49.
5. Zou W, Zhang DE. The interferon-inducible ubiquitin-protein isopeptide ligase (E3) EFP also functions as an ISG15 E3 ligase. The Journal of biological chemistry. 2005;281(7):3989-94.
6. Nakasato N, Ikeda K, Urano T, Horie-Inoue K, Takeda S, Inoue S. A ubiquitin E3 ligase Efp is up-regulated by interferons and conjugated with ISG15. Biochemical and biophysical research communications. 2006;351(2).
7. Catic A, Fiebiger E, Korbel GA, Blom D, Galardy PJ, Ploegh HL. Screen for ISG15-crossreactive deubiquitinases. PloS one. 2007;2(7).

**Reviewer #2:**

(Please note that all line numbers indicated below refer to the “tracked-changes” version of the manuscript).

1. Can the authors provide the results of statistical comparisons for the data in Figure 2F? The absence of any statistical test is conspicuous given that all other panels in every other figure include statistical comparisons.

Thank you for noticing this oversight. We have now added the statistical analysis to this panel.

2. Line 343: Please do not delete the word “with.” It is critical that the reader understands that VP35 was immunoprecipitated with TRIM25 (in the presence of NP), in reference to the left panel of Figure 5A. Removing the word changes the meaning of the sentence to imply that VP35 immunoprecipitated TRIM25, which is true, but only in the context of the right panel of Figure 5A. Given the discrepancy between the left and right panels, clarity is important here.

Corrected as suggested by the reviewer (line 330)

3. Line 363: The SPRY and RING/SPRY mutants do not appear to lose their interaction with NP. Instead, the interaction seems to be heavily reduced. The authors should revise this line to make the nuance clear. Similar revisions should be applied to Lines 402 and 583.

Corrected as suggested by the reviewer (lines 349, 398 and 595)

4. Lines 404: Similar to the above point, the 7KA mutation has not “lost” its interaction with NP. Instead, like the SPRY mutant, the interaction is reduced. Please revise this sentence (as well as line 583) to make the nuance clear.

Corrected as suggested by the reviewer (lines 398 and 595)

5. Did the authors conduct Western blots to evaluate protein expression and pull-down for the RNA IP assays depicted in Figure 7? The amount of RNA detected is likely directly related to the amount of protein precipitated, so it is important to know that sufficient and equal levels of protein were expressed and precipitated.

We have conducted protein expression analysis for the RNA IP assays at the time. However, we often obtained low quality blots, particularly for the NP pull-down samples. This is likely because the NP present is derived from incoming trVLPs (i.e., no NP overexpression) and the inclusion in the experimental setup of an unreversible UV-crosslinking. Due to these, together with the space constraints on Figures 7 and associated Figure S4, we initially decided to not include them.

Still taking into account some of the above considerations, we now show representative blots of our most relevant RNA IPs where is clearly shown that incoming NP was expressed and pulled down in equivalent amounts. Similar analysis is also shown for TRIM25 on those experiments (Figures S4E, S4H and S4I). We also added the levels of expressed and pulled-down ZAP, as well as incoming NP levels, to Figure 7G which is related to ZAP-associated trVLP genome.

6. Lines 468 and 477: The authors may want to avoid the use of the term “infection” in this context. While the intent may be clear to some readers, others may not fully grasp that trVLPs are inherently not infectious. Moreover, this particular experiment should not be construed as a replacement for conducting confirmatory experiments with authentic EBOV.

We use now the term “transduction” to avoid any misunderstanding (line 471 and 486).

**Reviewer #3:** (No Response)
